# Glycerol‐3‐phosphate acyltransferase 6 controls filamentous pathogen interactions and cell wall properties of the tomato and *Nicotiana benthamiana* leaf epidermis

**DOI:** 10.1111/nph.15846

**Published:** 2019-05-10

**Authors:** Stuart Fawke, Thomas A. Torode, Anna Gogleva, Eric A. Fich, Iben Sørensen, Temur Yunusov, Jocelyn K. C. Rose, Sebastian Schornack

**Affiliations:** ^1^ Sainsbury Laboratory (SLCU) University of Cambridge Cambridge UK; ^2^ Plant Biology Section School of Integrative Plant Science Cornell University Ithaca NY USA

**Keywords:** cell wall, cuticle, haustoria, *Nicotiana benthamiana*, oomycetes, *Phytophthora*, *Solanum lycopersicum*, stomata

## Abstract

- The leaf outer epidermal cell wall acts as a barrier against pathogen attack and desiccation, and as such is covered by a cuticle, composed of waxes and the polymer cutin. Cutin monomers are formed by the transfer of fatty acids to glycerol by glycerol‐3‐phosphate acyltransferases, which facilitate their transport to the surface.
- The extent to which cutin monomers affect leaf cell wall architecture and barrier properties is not known. We report a dual functionality of pathogen‐inducible *GLYCEROL‐3‐PHOSPHATE ACYLTRANSFERASE 6* (*GPAT6*) in controlling pathogen entry and cell wall properties affecting dehydration in leaves.
- Silencing of *Nicotiana benthamiana NbGPAT6a* increased leaf susceptibility to infection by the oomycetes *Phytophthora infestans* and *Phytophthora palmivora*, whereas overexpression of *NbGPAT6a‐GFP* rendered leaves more resistant. A loss‐of‐function mutation in tomato *SlGPAT6* similarly resulted in increased susceptibility of leaves to *Phytophthora* infection, concomitant with changes in haustoria morphology. Modulation of *GPAT6* expression altered the outer wall diameter of leaf epidermal cells. Moreover, we observed that tomato *gpat6‐a* mutants had an impaired cell wall–cuticle continuum and fewer stomata, but showed increased water loss.
- This study highlights a hitherto unknown role for GPAT6‐generated cutin monomers in influencing epidermal cell properties that are integral to leaf–microbe interactions and in limiting dehydration.

## Introduction

Most epidermal cells of the aerial parts of vascular plants are covered by a hydrophobic extracellular lipid barrier, known as the cuticle, which is composed of polymeric cutin and waxes (Yeats & Rose, 2013). The cutin matrix is a highly viscoelastic polymer with low tensile strength (Fich *et al*., 2016) that functions as a transpiration barrier (Schonherr, 1976) and also contributes mechanical strength to the underlying cell wall (Kolattukudy, 1980). Celluloses, hemicelluloses and pectins from the cell wall can be incorporated into the cutin matrix, thereby influencing its elasticity and stiffness (López‐Casado *et al*., 2007) and facilitating expansion during growth and development and in response to environmental cues (Bargel & Neinhuis, 2005; Underwood, 2012).

Cutin biosynthesis involves the esterification of oxygenated 16‐ or 18‐carbon fatty acids to glycerol (Beisson *et al*., 2012) through the action of glycerol‐3‐phosphate acyltransferases (GPAT4, GPAT6 and GPAT8). These enzymes have specificity for the second carbon of the glycerol (sn‐2 position) (Yang *et al*., 2012) and exhibit phosphatase activity that removes the phosphate group from glycerol‐3‐phosphate (Yang *et al*., 2010). Accordingly, mutants of the *Arabidopsis thaliana GPAT4*,* GPAT6* and *GPAT8* genes display reduced amounts of C16 and C18 fatty acid cutin monomers (Li *et al*., 2007; Mazurek *et al*., 2016). *GPAT4* orthologues in *Brassica napus* are highly expressed in the seed coat, periderm and endodermis of roots (Chen *et al*., 2011b) and function in the development of reproductive organs (Chen *et al*., 2014). *GPAT6* is involved in cutin synthesis in *A. thaliana* petals (Li‐Beisson *et al*., 2009) and tomato (*Solanum lycopersicum*) fruit (Petit *et al*., 2016) and was found to have multiple functions in stamen development and fertility (Li *et al*., 2012). Analysis of *A. thaliana gpat6* knockout lines demonstrated that GPAT6 is essential for the accumulation of C16 cutin monomers (Li‐Beisson *et al*., 2009) and that the enzyme has a higher affinity for C16 and C18 ω‐oxidized acyl‐CoA substrates. A glossy fruit mutant of the tomato cv Micro‐Tom with increased total wax load, but lower amounts of total cutin in fruit cuticles, and a much thinner cuticle (Petit *et al*., 2014), were discovered to be a result of a point mutation in the *GPAT6* gene that abolished enzymatic activity (Petit *et al*., 2016). Additionally, the Micro‐Tom *gpat6‐a* has perturbed pollen formation but is not male sterile (Petit *et al*., 2016).

The cuticle not only controls solute and gas exchange (Kerstiens, 1996a; Riederer & Schreiber, 2001) but also provides protection against pathogen invasion (Kerstiens, 1996a,b). Accordingly, to gain entry into host tissues, pathogens secrete hydrolytic enzymes, including cutinases, esterases, lipases and glycanases, which destroy the integrity of the cuticle–cell wall continuum (Belbahri *et al*., 2008; Blackman *et al*., 2014). For example, the oomycete *Phytophthora infestans* secretes cell wall‐ and cuticle‐degrading enzymes and forms surface appressoria that support tissue invasion. *P. infestans* is an economically important leaf pathogen of potato (*Solanum tuberosum*) and tomato (Haverkort *et al*., 2008) and can also infect wild tobacco species, including *Nicotiana benthamiana* (Becktell *et al*., 2006). During the early infection stages, *P. infestans* lives as a biotroph, proliferates an extensive intercellular hyphal network within the leaf mesophyll and projects short digit‐like haustoria into mesophyll cells to suppress immunity and support infection. In the later stages of infection, *P. infestans* switches to a necrotrophic lifestyle and kills the host tissue, resulting in necrotic lesions. Other *Phytophthora* species with similar lifestyles are not restricted to infecting aerial tissues. For example, the tropical pathogen *P. palmivora* can infect roots and shoots of many vascular and nonvascular host plants (Torres *et al*., 2016).

Cutin monomers and cell wall oligosaccharides released during a pathogen attack can serve as damage‐associated molecular patterns, allowing the plant cell to mount mitigating defence and wall repair responses, but conversely may also stimulate pathogen colonization by triggering the formation of appressoria (Gilbert *et al*., 1996). *Phytophthora palmivora* forms appressoria when exposed to cutin monomers *in vitro* (Wang *et al*., 2012) and cutin components have also been reported to trigger spore germination and cutinase expression in the fungus *Botrytis cinerea* (Leroch *et al*., 2013). The ectopic application of cutin monomers during root inoculation with *P. palmivora* was reported to restore full susceptibility to the *Medicago truncatula* GPAT mutant *ram2* (Wang *et al*., 2012), suggesting that the oomycete in part relies on the presence of cutin‐derived signals to enhance its pathogenicity, as reducing GPAT activity enhances resistance to *Phytophthora* infection.

Here we document the importance of GPAT6 in leaf infections by oomycete and fungal pathogens, as well as its contribution to cell wall properties. We found that *GPAT6* transcript abundance increases in response to *Phytophthora* infection, and that overexpression of *GPAT6* results in increased resistance to oomycete infection. Furthermore, although *gpat6* mutants are more susceptible to *Phytophthora* leaf infection, they display increased leaf resistance to *B. cinerea*, suggesting pathogen lifestyle‐specific differences. Changes in pathogen susceptibility are associated with altered thickness of the leaf cell wall plus cuticle, as well as altered transpiration and numbers of stomata. This is reflected in elevated transcript abundance of the immunity‐ and stomata development‐associated receptor‐like kinases *SERK3*/*BAK1* and *ERECTA* in *gpat6‐a* leaves. Cuticle‐associated genes are consistently altered in leaves and fruits of *gpat6‐a* plants, whereas more variation exists in genes related to the cell wall and secondary metabolites. Although *GPAT6*‐like genes have been implicated in flower, fruit and seed development, our work uncovers a function in leaves of *N. benthamiana* and tomato where *GPAT6* genes influence cell wall and cuticular properties associated with pathogen infection and water regulation.

## Materials and Methods

### Statistical analysis

Levene's tests were applied to check for heteroscedasticity between treatment groups. Following this, the appropriate two‐sample *t*‐test was applied, accounting for equal or unequal variances, to assess whether the means of two different treatment groups were significantly different, based on *α* = 0.5. Figures are labelled with asterisks to indicate *P*‐value range (i.e. *, *P* ≤ 0.05; **, *P* ≤ 0.01; ***, *P* ≤ 0.001).

### Microbial strains and cultivation

*Phytophthora infestans* strain 88069, previously described in van West *et al*. (1998), was grown at 18°C in the dark on rye sucrose agar plates. Zoospores were harvested from 14‐d‐old plates by adding 6 ml cold sterile H_2_O, incubating in the dark at 4°C for 45–60 min, then in the light at room temperature for 30 min and extracting the liquid by pipetting. Approximately 30 spores μl^−1^ sterile H_2_O were then used immediately for infection assays.

*Phytophthora palmivora* strain P16830‐YKDEL was previously described (Rey *et al*., 2013). *P. palmivora* was grown in a Conviron (Winnipeg, MB, Canada) A1000 Reach‐In Plant Growth Chamber at 25°C and 700 μmol intensity. For subculturing, rye sucrose agar plates were used with the addition of 50 μg ml^−1^ G418 (geneticin) to select for transformants. For production of zoospores, agar plates containing 10% unclarified V8 vegetable juice were used with the addition of 50 μg ml^−1^ G418 (geneticin). Harvesting of zoospores was performed as for *P. infestans* described earlier

*Botrytis cinerea* R190/11/3, isolated from *Geranium* by Robert Saville in 2011 (NIAB‐EMR, East Malling, UK) was grown on potato dextrose agar plates in a Conviron A1000 Reach‐In Plant Growth Chamber at 25°C and 700 μmol intensity and subcultured by excising an agar plug containing conidiophores and inverting it onto a fresh plate. Conidia for infection assays were harvested from 7‐d‐old potato dextrose agar plates but adding 6 ml cold sterile H_2_O, incubating in the light at room temperature for 1 h then gently agitating the conidiophores with a spatula to release the conidia. The concentration was adjusted to *c*. 30 conidia μl^−1^ sterile H_2_O.

### Leaf infection assays

Droplets (10 μl) of identical *Phytophthora* zoospore (*c*. 1000 zoospores) or *Botrytis* conidia count (*c*. 5000 spores) were placed onto the abaxial side of all leaves of the experiment between their veins. Inoculated leaves were then incubated in a humidified chamber under illumination at 24°C for the stated time before imaging. Disease symptom images of *P. infestans* or *P. palmivora* lesions were obtained by placing the infected leaves on a light table emitting near‐UV light in the 380–500 nm range and covering them with a yellow filter. This created high contrast between uninfected zones that appear red/orange as a result of autofluorescence and infected zones that appear black as a result of cell death‐associated loss of autofluorescence. Leaves were imaged through the filter using a digital camera on a tripod with long exposure settings and lesion area was quantified using imagej. Quantification involved conversion to greyscale, inversion of the colours then application of a threshold (same threshold for all images) to isolate the nonautofluorescent necrotic lesions from autofluorescent background. Nonautofluorescent pixels were counted by making a selection around all of the lesions on a single leaf and then using the ‘Analyze Particles’ function to calculate the total necrotic area per leaf.

### Phylogenetic analysis

Protein sequences from *N. benthamiana*,* M. truncatula* and *S. lycopersicum* homologous to *A. thaliana* GPATs were identified by search of the NCBI database (https://blast.ncbi.nlm.nih.gov/Blast.cgi) using AtGPATs 1–9 as queries (sequence accession numbers are listed in Supporting information Fig. S1). Obtained sequences were then aligned using muscle (http://www.ebi.ac.uk/Tools/msa/muscle/) and a phylogenetic tree constructed using phyml (http://atgc.lirmm.fr/phyml/) with the Phylogeny.fr web tool (http://www.phylogeny.fr/). Branches with < 50% bootstrap support (100 iterations) were collapsed. The tree presented in Fig. S1 was rendered using treedyn (http://www.treedyn.org/) and annotated using gimp (https://www.gimp.org/).

### Confocal microscopy

Confocal microscopy was performed using a Leica SP8 (Wetzlar, Germany) equipped with a white light laser (main laser power 70%, time gating) and a 63× water immersion objective and the following settings: pinhole, 1.00 AU; scan speed, 200 min^–1^; line averaging factor, 4; green fluorescent protein (GFP) excitation, 489 nm; emission window, 500–552 nm; mCherry excitation, 587 nm; emission window, 596–643 nm; plastid autofluorescence excitation, 489 nm; emission window, 650–700. Samples were mounted in water. Images of subcellular localiZation of NbGPAT6a‐GFP protein fusion were taken at 48 h postinoculation upon transient constitutive expression in *N. benthamiana* leaf.

### Quantitative reverse transcription polymerase chain reaction (qRT‐PCR)

Total RNA was extracted from plant material using Qiagen RNeasy Plant Mini Kit (Qiagen), including 1% (v/v) β‐mercaptoethanol in the extraction buffer. RNA was then reverse‐transcribed to cDNA using the Roche Transcriptor First Strand cDNA Synthesis Kit. qPCR was performed in 384 well plates using a Roche LightCycler 480 SYBR Green I Master Mix in a Roche LightCycler 480 II machine. Three technical replicates were performed for each sample. Normalization of crossing point‐PCR‐cycle (Cp) values to an internal control was performed against *NbEF1a*,* NbF‐BOX* or *NbL23* (Liu *et al*., 2012) for quantification of *N. benthamiana* transcripts and against *PiWS21* (Yan & Liou, 2006) for *P. infestans* transcripts.

### Expression analysis

Leaves of 6‐wk‐old tomato cv ‘Micro‐Tom’ or *gpat6‐a* mutant plants were subjected to a detached leaf infection assay (see earlier) and either zoospore suspension or water were applied to the lower epidermis. Leaf discs were harvested 72 h postinoculation (hpi). Three biological replicates per sample were obtained and subjected to RNA extraction and poly(A) selection. cDNA library preparation was performed with the TruSeq^®^ RNA Sample Preparation Kit (Illumina, San Diego, CA, USA) according to the manufacturer's protocol. cDNA sequencing of the 12 samples was performed with Illumina NextSeq 2500 in 100 paired‐end mode (Genewiz, Leipzig, Germany). Raw reads were subjected to quality control with fastqc (https://www.bioinformatics.babraham.ac.uk/projects/fastqc/) and then aligned back to the *S. lycopersicum* reference genome ITAG3.1 (ftp://ftp.solgenomics.net/tomato_genome/annotation/ITAG3.1_release/) using star (v.2.5.2b) aligner. Raw counts were obtained with featurecounts (Liao *et al*. 2014), and only uniquely mapped and properly paired reads were considered further. Differentially expressed genes were identified with the deseq2 bioconductor package (Love *et al*. 2014) following four pairwise comparisons. Differentially expressed genes (absolute log fold‐change (LFC) ≥ 1.5 and adjusted *P*‐value ≤ 10^−3^) were used to generate volcano plots and upset plots using upsetr package (Conway *et al*., 2017).

### Cryoscanning electron microscopy

Cryoscanning electron microscopy (cryo‐SEM) was performed on 6‐wk‐old *N. benthamiana* and tomato leaves using a Zeiss EVO HD15 (Oberkochen, Germany) with a Quorum cryo‐prep deck and cryo‐stage (Lewes, UK). Leaf sections were mounted, frozen, positioned inside the cryo‐prep deck and then fractured using a blade to allow for cross‐sectional imaging. Sublimation of samples for 3 min was used to remove surface ice and a 5 nm platinum coating was applied before imaging via secondary electron detection.

#### Cell wall porosity analysis

Discs (5 mm diameter) were excised from leaves using a cork borer and incubated for 1 h at room temperature with tetramethylrhodamine isothiocyanate (TRITC) : Dextran (0.1 mg ml^−1^, 150k mw; TDB Consultancy AB, Uppsala, Sweden) and Auramine O (0.01% w/v; Sigma). Images were acquired using a Leica SP8 equipped with white light laser (TRITC: excitation, 561 nm; emission window, 609–631 nm; AuramineO: excitation, 458 nm; emission window, 485–532 nm).

#### Water loss/dehydration analysis

Whole leaves were harvested, placed in a ventilated oven (30°C; MAXQ‐6000, Thermo Fisher Scientific, Waltham, MA, USA) and weighed over a time course.

## Results

### *GPAT6* is induced during *Phytophthora* leaf infections

GPAT enzymes function in root interactions with symbiotic arbuscular mycorrhiza fungi and pathogenic *Phytophthora* oomycetes (Wang *et al*., 2012), but their roles in leaf interactions with pathogens have not been well characterized. To this end, we first identified all GPATs encoded in the tomato and *N. benthamiana* genomes and grouped them based on their phylogenetic relationship to the better characterized *A. thaliana* homologues (Fig. S1; Methods S1). This revealed the presence of three *N. benthamiana* genes grouping with *AtGPAT1/2/3* that probably contribute to storage lipid biosynthesis (Zheng *et al*., 2003), and three genes associated with *AtGPAT5/7* that may be involved in suberin biosynthesis (Beisson *et al*., 2007). The two clades associated with *AtGPAT4/8* and *AtGPAT6*, implicated in *A. thaliana* cutin biosynthesis (Li *et al*., 2007; Li‐Beisson *et al*., 2009), contain two *N. benthamiana* genes each (Fig. S1). An additional group of six *N. benthamiana* genes form a clade together with *MtRAM2* that is distinct from any *A. thaliana GPAT* genes, and were termed *NbRAM2A‐F*.

**Figure 1:**
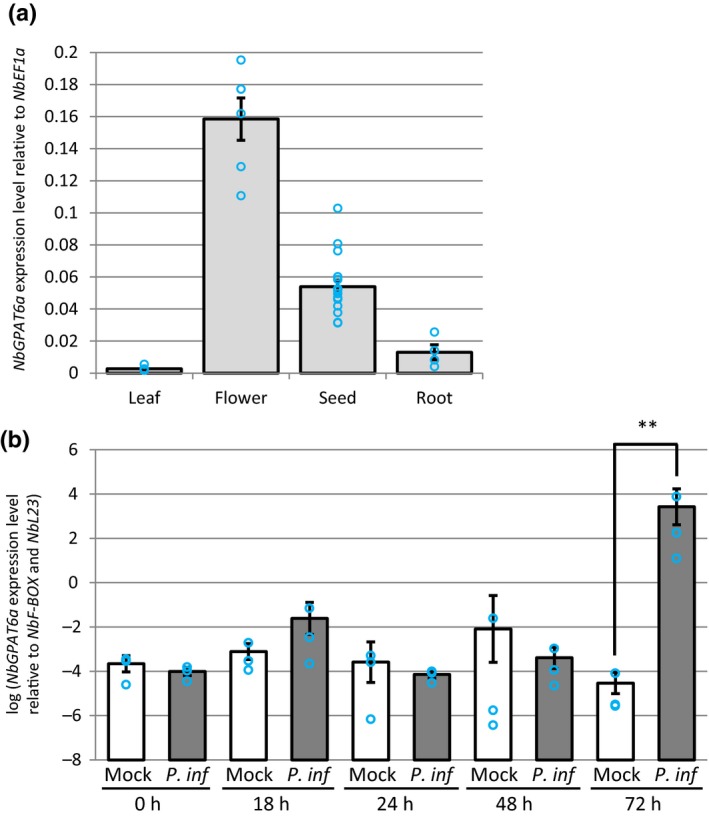
*NbGPAT6a* transcript abundance varies between *Nicotiana benthamiana* plant organs and is upregulated during *Phytophthora infestans* (*P. inf*) leaf colonization. (a) Expression level of *NbGPAT6a* in leaf, flower, seed and root tissues. Error bars represent ± SE of the mean of at least three biological replicates. Blue circles represent the average of three technical replicates. (b) Expression level of *NbGPAT6a* in leaf tissues is significantly increased at 72 h after inoculation with *P. infestans*. Error bars represent ± SE of the mean of three biological replicates (**, *P *<* *0.01). Blue circles represent the average of three technical replicates.

Previously published data on *N. benthamiana* root infection by *P. palmivora* reported that *NbGPAT6a*, but none of the other members of the GPAT4/6/8 clade, showed a consistent and significant transcriptional induction during all stages of infection (Fig. 1; Table S1) (Evangelisti *et al*., 2017).

Using qRT‐PCR, we detected *NbGPAT6a* expression in leaves, flowers, seeds and roots, with the highest steady‐state levels in flowers (Fig. 1). This is in agreement with *GPAT6* expression patterns in other species (Li‐Beisson *et al*., 2009; Li *et al*., 2012; Petit *et al*., 2016).

To test whether *NbGPAT6a* expression levels increase during infection, we infected *N. benthamiana* leaves with *P. infestans* strain 88069 (van West *et al*., 1999) zoospore droplets and measured changes in *NbGPAT6a* expression over time. *NbGPAT6a* was highly induced in leaf tissues at 72 hpi with *P. infestans* (Fig. 1). We therefore conclude that *NbGPAT6a* expression is upregulated in roots and leaves infected with *Phytophthora* and that expression levels in leaves are elevated late during infection and so are not part of early, inducible, defence responses.

### Constitutive expression of *NbGPAT6a* renders leaves resistant to *Phytophthora* infection

**Figure 2:**
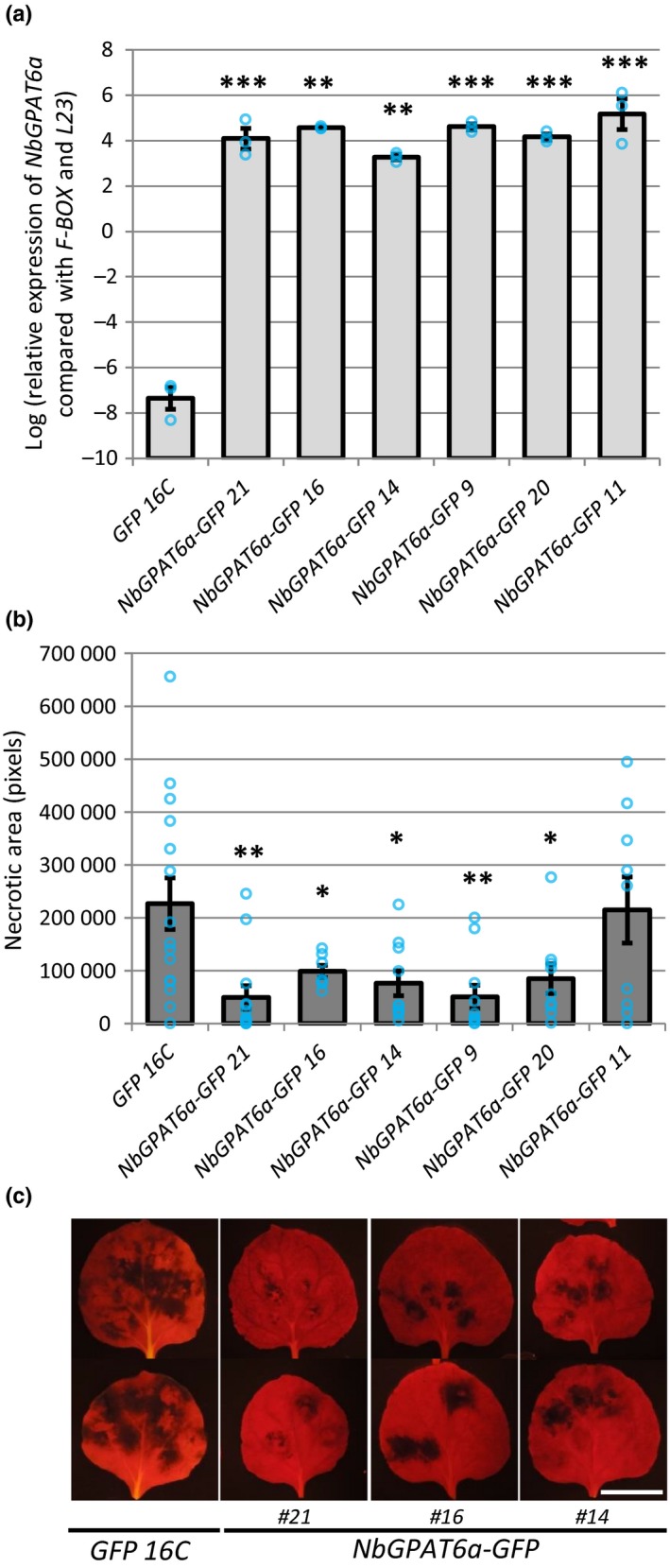
Constitutive overexpression of *NbGPAT6a* renders *Nicotiana benthamiana* leaves more resistant to infection by *Phytophthora infestans*. (a) Confirmation of *NbGPAT6a‐GFP* overexpression in transgenic *N. benthamiana* leaves, compared with control plants constitutively expressing *GFP* alone, quantified by quantitative reverse transcription polymerase chain reaction (qRT‐PCR). Error bars represent ± SE of the mean of three biological replicates. Significant differences are indicated as: *, *P* < 0.05; **, *P* < 0.01; ***, *P* < 0.001. Blue circles represent the average of three technical replicates. (b) Quantification of necrotic area present on leaves constitutively expressing *NbGPAT6a‐GFP* compared with the control expressing *GFP* alone following inoculation with *P. infestans*. Error bars represent ± SE of the mean of at least seven leaves from each transgenic line. Blue circles represent necrotic area on each leaf. (c) Representative images of leaves inoculated with *P. infestans* and imaged under blue light illumination using a yellow filter to record plastid red autofluorescence and to quantify necrotic area. Scale bar, 30 mm.

To address whether higher *GPAT6a* transcript abundances influence *Phytophthora* infection, we generated constitutive overexpression constructs by creating a translational fusion of the genomic *NbGPAT6a* open reading frame to the *GFP* reporter gene under control of the 35S promoter. We first investigated the subcellular distribution of the fusion protein upon transient expression in *N. benthamiana* leaves. GPAT6 is a predicted endoplasmic reticulum (ER)‐resident enzyme (Chen *et al*., 2011a) with two transmembrane domains (Fig. S2b) and we observed NbGPAT6‐GFP signals in the ER of leaf epidermal cells matching the subcellular distribution of ER‐targeted red fluorescent protein (RFP) (Fig. S2a). We then generated several independent *N. benthamiana* lines that stably and constitutively expressed *NbGPAT6a‐GFP* (Fig. 2).

Overexpression of *NbGPAT6a‐GFP* resulted in a 73% increase in total leaf cutin, which was almost entirely a result of elevated concentrations of ω‐hydroxyl (OH) fatty acid (‐FA) cutin monomers (Fig. S3a). In particular, concentrations of hexadecane‐dioic acid, ω‐hydroxy hexadecanoic acid, ω‐hydroxy heptadecanoic acid, ω‐hydroxy‐octadecanoic acid and 10,16‐dihydroxy hexadecanoic acid showed significant increases relative to GFP16C (control) leaves (Fig. S3b).

When we tested *NbGPAT6a‐GFP* transgenic plants for their resistance to *P. infestans* leaf infections, we found that five of the six lines displayed smaller necrotic areas than the control lines (Fig. 2) without affecting overall morphology of *P. infestans* hyphae or haustoria within leaf epidermal cells of two independent *NbGPATa‐GFP* transgenic lines (Fig. S4). Notably, transient expression of *NbGPAT6a‐GFP* in fully expanded leaves followed 24 h later by *P. infestans* infection did not alter the extent of disease‐associated leaf necrosis (Fig. S5), suggesting that NbGPAT6‐mediated resistance is associated with longer‐term leaf development processes. Variation in these leaf development processes may contribute to the variation in resistance phenotype we observed across the six independent overexpressing lines.

### Knockdown or knockout of *GPAT6* renders leaves more susceptible to *Phytophthora* infection but more resistant to *B. cinerea*

**Figure 3:**
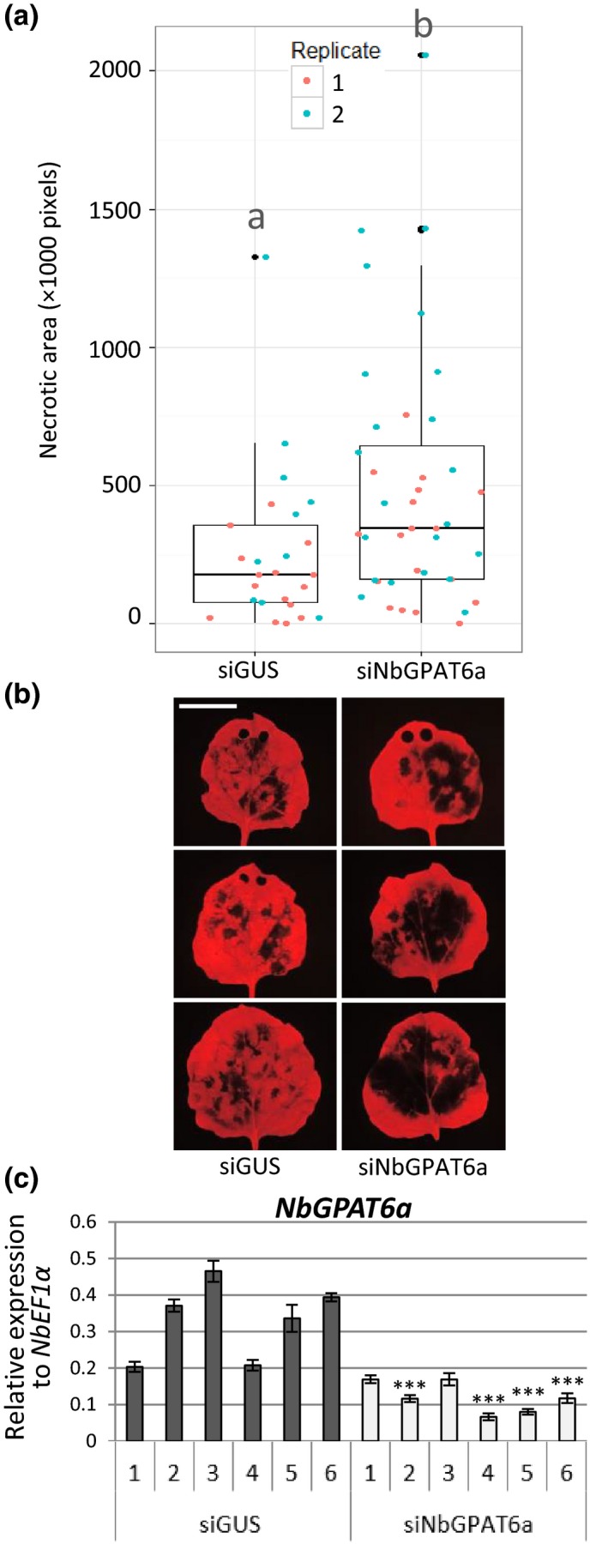
siNbGPAT6a‐mediated virus‐induced gene silencing (VIGS) results in stronger *Nicotiana benthamiana* leaf necrosis upon *Phytophthora infestans* infection. (a) Leaf necrotic area 5 d postinoculation (dpi) with *P. infestans*, as quantified by lack of red Chl fluorescence, two replicates combined (*P *=* *0.0127). Data points for each replicate are denoted by different colours. Horizontal lines represent median and upper and lower quartiles. Whiskers extend to data points that are < 1.5 × interquartile range away from upper/lower quartile. Lower case letters ‘a’ and ‘b’ indicate significant differences between the means as compared using Student's *t*‐test (*P* < 0.05). (b) Representative UV images of leaf necrosis quantified in (a). Scale bar, 30 mm. Holes at the leaf tip in some images are the result of tissue samples taken for quantification of transcript abundance (these areas were excluded from necrotic area quantification). (c) Relative expression of *NbGPAT6a* (to *NbEF1α*) following VIGS using siNbGPAT6a or siGUS (control). Error bars represent ± SE of the mean of three biological replicates. Student's *t*‐test was used to compare mean relative expression in siNbGPAT6a samples with the lowest of control samples. *, *P *<* *0.05; **, *P *<* *0.01; ***, *P *<* *0.001.

To test whether reduced *GPAT6* levels cause the opposite phenotype to increased levels, we established a *NbGPAT6a*‐specific virus‐induced gene silencing (VIGS) construct and demonstrated that it attenuated transcript abundances of *NbGPAT6a*, but not those of homologous transcripts (Figs 3, S6). We found that siNbGPAT6a‐mediated VIGS resulted in stronger leaf necrosis upon *P. infestans* infection (Fig. 3), suggesting a higher degree of susceptibility.

**Figure 4:**
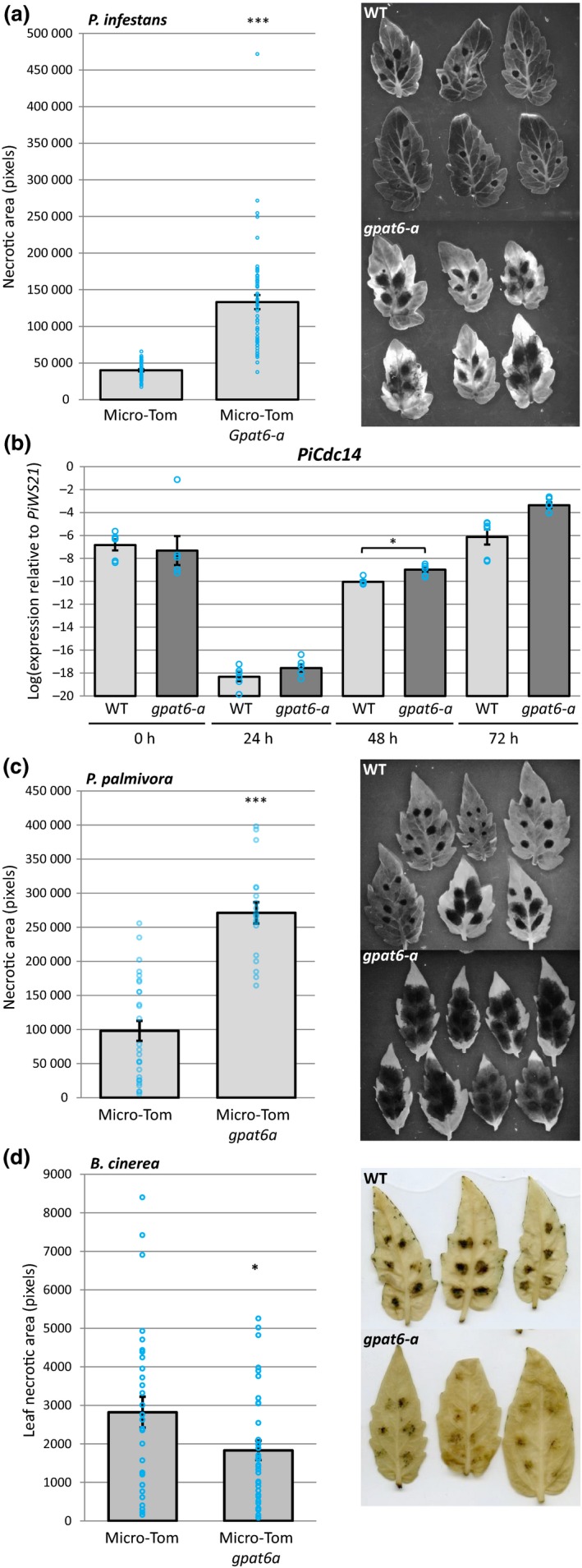
Tomato *gpat6a* mutants are more susceptible to *Phytophthora infestans* and *Phytophthora palmivora* infection but more resistant to *Botrytis cinerea* infection. (a) Quantification of Micro‐Tom leaf necrotic area at 72 h postinoculation with *P. infestans* for both wild‐type and *gpat6‐a* genotypes. Error bars represent ± SE of the mean. Each blue circle indicates the sum of necrotic area for one leaf, four inoculation sites. Representative UV images of wild‐type (WT) and *gpat6‐a* leaves used for necrotic area quantification are also shown. (b) Expression level of the sporulation‐specific *PiCdc14* (sporulation marker) normalized to *PiWS21* (40S ribosomal protein S3A). Error bars represent ± SE of the mean of three biological replicates (six technical replicates indicated by blue circles). *, *P* < 0.05. (c) Quantification of Micro‐Tom leaf necrotic area at 72 h postinoculation with *P. palmivora* for both WT and *gpat6‐a* genotypes. Errors bars represent ± SE of the mean (*n* = 27 for WT, *n* = 19 for *gpat6‐a*). Each blue circle indicates the sum of necrotic area for one leaf, six inoculation sites. ***, *P* < 0.001. (d) Quantification of leaf necrotic area at 6 d postinoculation with *B. cinerea*. Blue circles represent the total necrotic area for each leaf (six droplets of *B. cinerea* spore solution). Error bars represent ± SE of the mean (*n* = 30 for WT, *n* = 35 for *gpat6‐a*). Representative images of leaves used to quantify leaf necrotic area are also shown. This experiment was repeated twice with similar results.

We next tested whether GPAT6 contributes to *Phytophthora* infection in tomato using a *gpat6‐a* mutant in the ‘Micro‐Tom’ background (Petit *et al*., 2016). Detached leaf infection assays showed that the tomato *gpat6‐a* mutant was more susceptible to *P. infestans* (Fig. 4a,b) and *P. palmivora* infection (Fig. 4c), as evident by larger lesion sizes and higher expression levels of *P. infestans* sporulation marker transcript abundances at 48 hpi. When investigating infection structures, we found that *P. infestans* formed normal, digit‐like haustoria (55%) but also singly branched haustoria (45%) in epidermal cells of *gpat6‐a* mutant tomato leaves (65 haustoria counted; Fig. S7a). By contrast, almost exclusively digit‐like haustoria (92%, 61 haustoria counted) were formed in wild‐type (WT) leaves (Fig. S7b). Importantly, *gpat6‐a* mutants displayed less severe disease symptoms upon infection with the fungal pathogen *B. cinerea* (Fig. 4d). Taken together these data demonstrate that attenuating or knocking out the expression of *GPAT6* genes has the opposite effect to *GPAT6* gene overexpression, further supporting an important role for GPAT6 in ensuring full resistance to *Phytophthora* infections.

### Modulating *GPAT6* expression alters the thickness of the outer cell walls of the leaf epidermis

**Figure 5:**
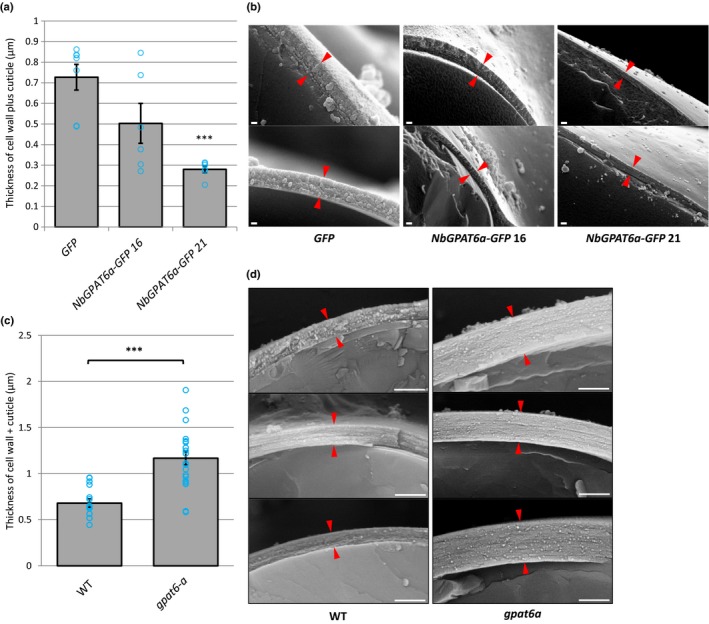
Outer epidermal cell wall thickness is correlated to *GPAT6* expression levels. (a) Quantification of cell wall plus cuticle thickness in *Nicotiana benthamiana* leaves constitutively expressing *NbGPAT6a‐GFP*. Blue circles represent the mean of three measurements per image (*n* = 7 for *GFP*,* n* = 6 for *NbGPAT6a‐GFP* 16 and *NbGPAT6a‐GFP* 21). Error bars represent ± SE of the mean. ***, *P *<* *0.001. (b) Representative cryoscanning electron microscopy (cryo‐SEM) images of transverse fractures used to quantify thickness. Red arrowheads indicate the boundary of cell wall plus cuticle. Scale bars, 200 nm. (c) Quantification of cell wall plus cuticle thickness in leaves of tomato *gpat6‐a* mutants. Blue circles represent the mean of three measurements per image (*n* = 15 for wild‐type (WT), *n* = 20 for *gpat6‐a*). Error bars represent ± SE of the mean. ***, *P *<* *0.001. (d) Representative cryo‐SEM images of transverse fractures used to quantify thickness. Red arrowheads indicate the boundary of cell wall plus cuticle. Scale bars, 1 μm. This experiment was repeated twice with similar results.

The tomato *gpat6‐a* mutant was reported to have an altered fruit cuticle structure (Petit *et al*., 2016), which we hypothesized might be associated with the altered *Phytophthora* infection phenotypes described earlier. We imaged the cell wall and cuticle of *NbGPAT6‐GFP N. benthamiana* leaves that displayed different degrees of resistance to *P. infestans* infection, as well as tomato *gpat6‐a* mutant leaves, using cryo‐SEM. We observed that the outer epidermal cell wall was thinner in *NbGPAT6a‐GFP* lines compared with those expressing GFP alone (Fig. 5a,b), particularly in the line showing the highest *P. infestans* resistance (*NbGPAT6a‐GFP* #21).

Conversely, *gpat6‐a* leaf epidermal cells possessed a thicker cell wall (Figs 5c,d, S8a–e). This change in thickness was most prominent in the outer, but not the inner, periclinal wall of both the abaxial and adaxial leaf epidermis (Fig. S8e). Thus, the cell wall thickness inversely correlated with the level of *GPAT6* expression.

**Figure 6:**
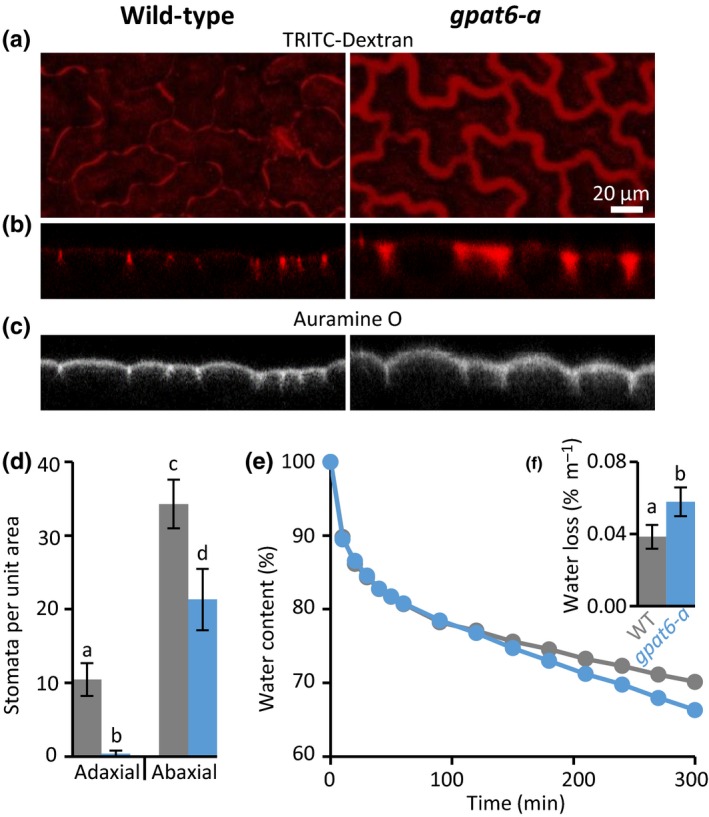
Leaf outer epidermal walls of tomato *gpat6‐a* mutant display altered porosity, reduced numbers of stomata and increased susceptibility to desiccation. Panels (a)–(c) are of identical magnification represented by the scale bar in (a). TRITC‐Dextran (150 kDa) distribution imaged from above (a) and confocal transects (b) are extended in *gpat6‐a* mutant epidermal walls. Auramine O was used to label cutin (c). (d) Stomatal numbers per unit leaf area; (e, f) water loss over time and total water loss. Error bars represent ± SD of six biological replicates. Different lowercase letters above error bars indicate significant difference between means.

GPAT6 enzymes are known to be involved in cutin biosynthesis (Li‐Beisson *et al*., 2009) and *gpat6‐a* tomato fruits have increased cuticle permeability to the dye toluidine blue. We found that *gpat6‐a* leaves did not show significantly altered permeability when toluidine blue was placed on the upper or lower epidermis, whereras abrasive treatment with bentonite/cellite resulted in full permeability, confirming the suitability of our staining procedure (Fig. S9a,b). To test for changes in wall porosity, we applied Dextran‐150 kDa‐TRITC to WT and *gpat6‐a* epidermis. Subsequent fluorescent imaging showed that TRITC‐labelled Dextran was incorporated to a greater extent in the *gpat6‐a* mutant, suggesting a larger porosity of the wall (Fig. 6).

Concomitantly, *gpat6‐a* tomato leaves showed an increased rate of water loss compared with the WT (Fig. 6e,f). This was significant in both the total amount of water loss and the relative water loss over time. We also observed that the *gpat6‐a* mutant leaves had fewer stomata than the WT (Figs 6d, S10a), but that the numbers increased to a value similar to the WT when *gpat6‐a* plants were grown under high humidity conditions (Fig. S10b). We did not observe changes in stomata numbers (Fig. S11a) or water loss over time in overexpressing *N. benthamiana GPAT6a‐GFP* lines with thinner walls (Fig. S11b,c), suggesting that the cuticle permeability was not altered, even though it was thinner. Furthermore, our analysis of the composition and overall architecture of the bulk leaf cell wall using cell wall antibodies did not reveal any significant alterations in *GPAT6‐GFP*‐overexpressing plants or the *gpat6‐a* mutant (Fig. S12).

### The *gpat6‐a* leaf transcriptome reflects changes in cuticle and cell wall processes and stomatal patterning

**Figure 7:**
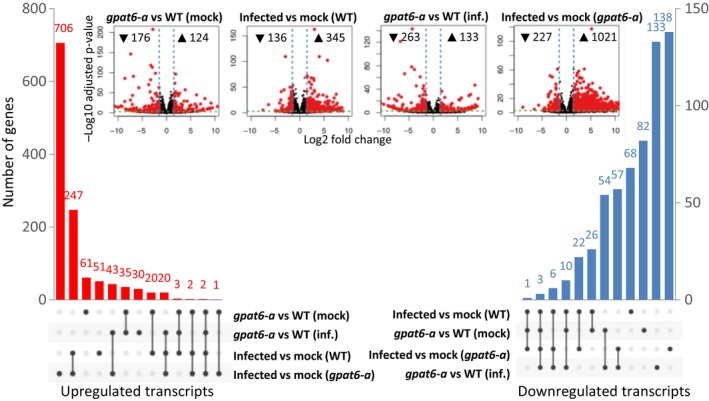
Transcriptome differences in *gpat6‐a* vs wild‐type (WT) tomato cv ‘Micro‐Tom’ leaves under control and infection conditions. Water or *Phytophthora infestans* 88069 zoospores was applied to detached leaves and harvested at 72 h postinoculation. Differentially upregulated (red) and downregulated (blue) transcripts (absolute log fold‐change ≥ 1.5 and adjusted *P*‐value ≤ 10^−3^) and pairwise comparison volcano plots (inset) are shown. This figure is based on Supporting Information Dataset S1.

To determine the impact of the *gpat6‐a* mutation on the tomato transcriptome, we carried out expression analysis of *gpat6‐a* and WT leaves from both *P. infestans*‐infected and control plants (Fig. 7), and compared our findings with previous expression data derived from the tomato fruit exocarp (Petit *et al*., 2016).

Considering a LFC ≥ 1.5 with an adjusted *P*‐value of significance < 10E–3, we found the expression of 124 genes to be upregulated and 176 to be downregulated compared with uninfected leaves of *gpat6‐a* and WT plants (Fig. 7, Dataset S1). Infection of *gpat6‐a* plants resulted in a much higher number of induced genes (1021) compared with infection in WT leaves (345), congruent with *gpat6‐a* leaves being much more susceptible to *P. infestans* infection. Notably, there was a higher proportion of genes associated with immune responses (Pombo *et al*., 2014) induced in *gpat6‐a* leaves (Dataset S1). The differences in downregulated genes were more moderate, with 227 in *gpat6‐a* compared with 136 in WT‐infected plants.

Petit *et al*. (2016) highlighted 42 genes associated with lipid, secondary metabolite, and cell wall biosynthesis as differentially expressed in the *gpat6‐a* fruit exocarp. We found only 29 of these genes to be altered in the same direction (Table S1). Genes belonging to the ‘cuticle’ category responded similarly in leaves and fruit exocarp, with 18 of 21 genes similarly differentially expressed in both organs. However, genes in the ‘cell wall’ gene category responded differently and although we found seven out of 10 also repressed in leaves, none of the five reported by Petit *et al*. (2016) was induced. Two genes, annotated as pectin methyl esterase inhibitor and cellulose synthase‐like, showed opposite expression dynamics between leaves and fruit. Petit *et al*. (2016) assigned six differentially expressed genes to the secondary metabolism category – three repressed and three induced. Of these, two were also repressed and induced in our dataset, and two were also induced. In summary, the expression levels of cuticle‐associated genes were consistently altered in leaves and fruits of *gpat6‐a* tomato plants. More variation was observed in the cell wall and secondary metabolite categories. As alterations in the cell wall were seen to be most extensive in the outer wall of the epidermis, this represents an interesting target for future studies of expression patterns. However, *P. infestans* infection rapidly disrupts epidermal integrity, which represents a major technical challenge.

Even in uninfected leaves, genes associated with defence and immunity, such as disease resistance genes, protease inhibitors, pathogenicity‐related genes (*PR‐3* and *PR‐5*), and chitinases were noted in the group of *gpat6‐a* repressed genes and absent from the group of induced genes (Dataset S1). Furthermore, seven genes encoding glutaredoxins were repressed in *gpat6‐a*, suggesting that this would increase susceptibility to redox stress. Together these data suggest that the increase in susceptibility of *gpat6‐a* can, in part, be attributed to constitutively lower levels of defence gene expression in uninfected *gpat6‐a* plants.

Six leucine‐rich repeat receptor like kinases (RLKs) were induced in *gpat6‐a* compared with WT plants (Dataset S1), including *SERK3/BAK1* (Solyc01g056655), which showed the strongest transcriptional upregulation of all genes differentially expressed between *gpat6‐a* and WT leaves, although defence‐associated genes were more frequently repressed in *gpat6‐a* mutants. A homologue of the *ERECTA* homologue (Solyc01g057680), which is associated with negative regulation of stomata density in *A. thaliana*, was also induced. The observed induction of both *SERK3/BAK1* and *ERECTA* may be related to the reduced number of stomata in *gpat6‐a* leaves. *A. thaliana* homologues of the other induced RLK encoding genes have been associated with pollen tube guidance (Solyc05g025780/LePRK3) (Gui *et al*., 2014), cell wall integrity sensing and resistance to *Fusarium oxysporum* root infections (Solyc01g014147, Solyc01g009930) (Van der Does *et al*., 2017) and regulating cell expansion through the transport of cell wall material (Solyc05g041750/PERK10) (Humphrey *et al*., 2014) but their roles in tomato have yet to be reported.

## Discussion

Previous studies have implicated *GPAT6* in the development of flowers and fruit, but its function in leaves has not been characterized. For example, *A. thaliana GPAT6* is highly expressed in flowers (more than two‐fold higher in petals and sepals than in other *GPAT* genes) and is known to function in stamen development and fertility (Li *et al*., 2012), while its homologue in tomato has an additional function in fruit cutin biosynthesis (Petit *et al*., 2016). We demonstrate that despite its low steady‐state expression levels, *GPAT6* fulfils an important role in leaves associated with epidermal outer cell wall properties that confer protection against dehydration, as well as infection by *Phytophthora* species.

### Late transcriptional upregulation of *NbGPAT6a* during *P. infestans* infection is a mitigation response

*Arabidopsis thaliana* GPAT6 is a *sn*2‐acyltransferase that is involved in cutin biosynthesis. Our analysis shows that *NbGPAT6a* overexpression results in increased amounts of cutin monomers (Fig. S3), indicative of a conserved function. The late transcriptional upregulation of *NbGPAT6* during *P. infestans* infection (Fig. 1) can be interpreted either as a pathogen‐controlled lipid harvesting strategy or, alternatively, as a mitigation response by the plant to tissue damage caused by pathogen colonization. It was recently hypothesized that obligate biotrophic fungal pathogens may exert a lipid parasitism, where the microbe benefits from plant fatty acid production (Jiang *et al*., 2017; Keymer & Gutjahr, 2018). In this context it was interesting to note a high frequency of aberrantly shaped haustoria in infected *gpat6‐a* tomato (Fig. S4). *P. infestans* haustoria are intracellular structures and characteristically digit‐shaped, although infrequently branched haustoria can also be observed (Blackwell, 1953). Whether an altered lipid metabolism in *gpat6‐a* tomato leaf epidermal cells might affect their development would require additional investigation using other lipid biosynthesis mutants. Nevertheless, *gpat6‐a* tomato mutants were more susceptible to both *P. infestans* and *P. palmivora* (Fig. 4), suggesting that the observed alteration in haustorium morphology does not impair infection and that the oomycete does not extensively rely on cutin monomers provided through GPAT6. It might also be informative to infect *gpat6‐a* tomato with obligate fungal pathogens, such as *Oidium neolycopersici*, to determine whether lipid parasitism is linked to an obligate biotrophic lifestyle. We conclude that *Phytophthora*‐controlled lipid harvesting is unlikely because overproduction of cutin monomers through overexpression of *NbGPAT6a‐GFP* did not result in increased pathogen‐caused symptoms; indeed, the leaves were more resistant to *P. infestans* infection (Fig. 2). Furthermore, *Phytophthora*‐derived cuticle and cell wall‐degrading enzymes probably release cutin monomers into the apoplast to enable sufficient uptake by the oomycete for continued infection in WT. We therefore propose that late transcriptional upregulation of *NbGPAT6a* during *P. infestans* infection is a mitigation response to tissue damage. Alternatively, it may be part of a delayed defence response, as various hydroxy fatty acid compounds have been implicated in pathogen resistance (Schweizer *et al*., 1996; Hou & Forman Iii, 2000; Wang *et al*., 2000), including against *P. infestans*.

### Differences in oomycete and fungal leaf infections may be attributable to their lifestyles

A range of mutations have been reported to increase both leaf cuticle permeability and pathogen susceptibility (Tang *et al*., 2007). However, these mutants are all more resistant to *B. cinerea* (Ziv *et al*., 2018). Explanations for this apparent paradox include an increased release of disease resistance activators, antifungal diffusible components and improved uptake of elicitors in the mutants (Tang *et al*., 2007; Ziv *et al*., 2018). We observed that constitutively expressing *NbGPAT6a* lines did not display altered leaf permeability and increased water loss (Fig. S11), suggesting that other processes contribute to their increased resistance. Conversely, *gpat6‐a* mutants showed reduced expression levels of defence‐ and stress‐associated genes (Dataset S1), which may influence the infection outcome. The ability of pathogens to infect these mutants may depend on the pathogen infection biology or lifestyle. *Phytophthora* pathogens are hemibiotrophs, which initially require living host cells for infection (Fawke *et al*., 2015), whereas *B. cinerea* is considered a necrotroph that immediately kills the tissue (Van Kan *et al*., 2017). This may explain why *gpat6‐a* tomato leaves exhibit resistance to *B. cinerea*. Whereas a reliance on plant lipid biosynthesis was recently demonstrated for an obligate biotrophic fungal pathogen (Jiang *et al*., 2017), this remains to be reported for oomycetes.

### The leaf cuticle layer may impose a physical restraint upon the outer facing epidermal cell wall

Our results suggest that GPAT6 influences various properties of the cell wall–cuticle superstructure. We show that loss of *GPAT6* increased the thickness of the wall (Figs 5c,d, S8), and overexpression of *NbGPAT6a‐GFP* led to higher amounts of cutin monomers and reduced wall thickness (Fig. 5a,b). Interestingly, this effect was mainly observed in the outer face of the epidermal walls, which are also the only walls associated with a cuticle (Fig. S8e), while the overall composition of the leaf bulk cell wall was not altered (Fig. S12). This suggests that the outer epidermal cell wall may respond to its ‘cutin status’ and adapt its thickness, possibly through mechanical or biochemical sensing. An instantaneous and reversible increase in thickness of the cell wall has previously been observed upon abrasion of the cuticle (Xia *et al*., 2009). This suggests that physical properties of the wall allow it to flexibly expand in diameter and that cutin monomers contribute to preventing excessive expansion. Similar increases in wall thickness were reported when a tomato cutin synthase was mutated, leading to a substantially thinner fruit cuticle (Yeats *et al*., 2012). Although we observed that *gpat6‐a* mutant leaves have a thicker cell wall and cuticle, whereas those of leaves constitutively expressing *NbGPAT6a* are thinner, there was not such a clear contrast in terms of the rate of water loss or stomata numbers. Specifically, *gpat6‐a* leaves lose more water over time than those of WT (Fig. 6e,f), whereas leaves constitutively expressing *NbGPAT6a* lose the same amount of water as the WT (Fig. S11). This suggests that a deficiency in cutin monomers during development has a significant impact on permeability, whereas an excess of cutin monomers can be tolerated or compensated for by the plant and has no effect on permeability.

The increased porosity of *gpat6‐a* mutants with thicker walls may result from the looser packing of wall components, or from the low amounts of cutin monomers in the wall. This, in turn, may cause the observed increased rate of water loss, which is compensated for by altering the number of stomata.

## Author contributions

SS, TAT, AG, SF and JKCR designed the research; SF, TAT, AG, EAF, IS, TY and SS performed research; SF, TAT, AG, EAF, IS, JKCR and SS analysed data; and SF, JKCR and SS wrote the paper.

## Supporting information

Please note: Wiley Blackwell are not responsible for the content or functionality of any Supporting Information supplied by the authors. Any queries (other than missing material) should be directed to the *New Phytologist* Central Office.

**Dataset S1** RNA‐seq of *Solanum lycopersicum* Micro‐tom WT and *gpat6a* leaves, infected with *Phytophthora infestans*.
**Fig. S1** Phylogenetic relationship of *N. benthamiana* GPAT‐related proteins to homologues in *M. truncatula*,* A. thaliana* and *S. lycopersicum.*
**Fig. S2** NbGPAT6a localizes to the endoplasmic reticulum and is predicted to have two transmembrane domains.
**Fig. S3** Cutin content of *NbGPAT6a‐GFP*‐expressing leaves is dramatically increased relative to *GFP 16C* (control) leaves.
**Fig. S4** *Phytophthora infestans* haustoria formed in wild‐type and NbGPAT6a‐GFP‐overexpressing *Nicotiana benthamiana* leaves are similar in structure.
**Fig. S5** Transient expression of *NbGPAT6a* does not alter leaf necrosis triggered by *P. infestans* infection.
**Fig. S6** Assessment of off‐target gene silencing following VIGS using siNbGPAT6a or siGUS (control).
**Fig. S7** *Phytophthora infestans* forms digit‐like and branched haustoria in tomato *gpat6‐a* mutants.
**Fig. S8** Thickness of outer cell wall plus cuticle is greater in *gpat6‐a* leaves than in the wild‐type.
**Fig. S9** Leaves of *gpat6‐a* tomato mutants are largely impermeable to Toluidine Blue.
**Fig. S10** Tomato *gpat6‐a* plants grown in water‐saturated atmosphere are not altered in their stomata numbers per leaf area.
**Fig. S11** Stomata numbers and water loss over time remain unaffected in *Nicotiana benthamiana* overexpressing *NbGPAT6‐GFP*.
**Fig. S12** Composition and overall architecture of the bulk leaf cell wall are not significantly altered in *GPAT6‐GFP*‐overexpressing plants or in the *gpat6‐a* mutant.
**Methods S1** Description of experimental materials and methods used to generate supplementary data.
**Table S1** Comparison of differentially regulated genes between WT and *gpat6a* Micro‐tom leaves identified by RNASeq in this study and that of Petit *et al*. (2016).

## Data Availability

The raw fastq data from the expression analysis are accessible at http://www.ncbi.nlm.nih.gov/sra/with accession number SRP158564. These data form the basis of Fig. 7 and Dataset S1. There are no restrictions on data availability.
