# Supplementary material for: Glycerol‐3‐phosphate acyltransferase 6 controls filamentous pathogen interactions and cell wall properties of the tomato and Nicotiana benthamiana leaf epidermis
Source: New Phytol. 2019 May 10;223(3):1547–59. doi: 10.1111/nph.15846 (PMC6767537; doi:10.1111/nph.15846)
Supplement: Supplementary file 1 — Dataset S1 RNA‐seq of Solanum lycopersicum Micro‐tom WT and gpat6a leaves, infected with Phytophthora infestans. Fig. S1 Phylogenetic relationship of N. benthamiana GPAT‐related proteins to homologues in M. truncatula, A. thaliana and S. lycopersicum. Fig. S2 NbGPAT6a localizes to the endoplasmic reticulum and is predicted to have two transmembrane domains. Fig. S3 Cutin content of NbGPAT6a‐GFP‐expressing leaves is dramatically increased relative to GFP 16C (control) leaves. Fig. S4 Phytophthora infestans haustoria formed in wild‐type and NbGPAT6a‐GFP‐overexpressing Nicotiana benthamiana leaves are similar in structure. Fig. S5 Transient expression of NbGPAT6a does not alter leaf necrosis triggered by P. infestans infection. Fig. S6 Assessment of off‐target gene silencing following VIGS using siNbGPAT6a or siGUS (control). Fig. S7 Phytophthora infestans forms digit‐like and branched haustoria in tomato gpat6‐a mutants. Fig. S8 Thickness of outer cell wall plus cuticle is greater in gpat6‐a leaves than in the wild‐type. Fig. S9 Leaves of gpat6‐a tomato mutants are largely impermeable to Toluidine Blue. Fig. S10 Tomato gpat6‐a plants grown in water‐saturated atmosphere are not altered in their stomata numbers per leaf area. Fig. S11 Stomata numbers and water loss over time remain unaffected in Nicotiana benthamiana overexpressing NbGPAT6‐GFP. Fig. S12 Composition and overall architecture of the bulk leaf cell wall are not significantly altered in GPAT6‐GFP‐overexpressing plants or in the gpat6‐a mutant. Methods S1 Description of experimental materials and methods used to generate supplementary data. Table S1 Comparison of differentially regulated genes between WT and gpat6a Micro‐tom leaves identified by RNASeq in this study and that of Petit et al. (2016). [file NPH-223-1547-s001.zip › nph15846-sup-0002-FigsS1-S12&MethodsS1.pdf]

New Phytologist **Supporting Information**

Article title: **Glycerol phosphate acyltransferase 6 controls filamentous pathogen interactions and cell wall properties of the tomato and *Nicotiana benthamiana* leaf epidermis**

Authors: Stuart Fawke, Thomas A. Torode, Anna Gogleva, Eric A. Fich, Iben Sørensen, Temur Yunusov, Jocelyn K.C. Rose, Sebastian Schornack

Article acceptance date: 29 March 2019

The following Supporting Information is available for this article:

Fig. S1 Phylogenetic relationship of *N. benthamiana* GPAT-related proteins to homologues in *M. truncatula*, *A. thaliana* and *S. lycopersicum.*

Fig. S2 NbGPAT6a localises to the endoplasmic reticulum and is predicted to have two transmembrane domains.

Fig. S3 Cutin content of *NbGPAT6a-GFP* expressing leaves is dramatically increased relative to *GFP 16C* (control) leaves.

Fig. S4 *Phytophthora infestans* haustoria formed in wildtype and NbGPAT6a-GFP overexpressing *Nicotiana benthamiana* leaves are similar in structure.

Fig. S5 Transient expression of *NbGPAT6a* does not alter leaf necrosis triggered by *P. infestans* infection.

Fig. S6 Assessment of off-target gene silencing following VIGS using siNbGPAT6a or siGUS (control).

Fig. S7 *Phytophthora infestans* forms digit-like and branched haustoria in tomato *gpat6-a* mutants.

Fig. S8 Thickness of outer cell wall plus cuticle is greater in *gpat6-a* leaves compared to wild-type.

Fig. S9 Leaves of *gpat6-a* tomato mutants are largely impermeable to Toluidine Blue.

Fig. S10 Tomato *gpat6-a* plants grown in water saturated atmosphere are not altered in their stomata numbers per leaf area.

Fig. S11 Stomata numbers and water loss over time remain unaffected in *Nicotiana benthamiana* overexpressing *NbGPAT6-GFP.*

Fig. S12 Composition and overall architecture of the bulk leaf cell wall is not significantly altered in *GPAT6-GFP* overexpressing plants or in *gpat6-a* mutant.

Methods S1 Description of experimental materials and methods used to generate supplementary data.

Dataset S1 (Supplementary_Dataset_1.xlsx) RNA-seq of *Solanum lycopersicum* Micro-tom WT and *gpat6a* leaves, infected with *Phytophthora infestans*. – separate document

Table S1 Comparison of differentially regulated genes between WT and *gpat6a* Micro-tom leaves identified by RNASeq in this study and that of Petit et. al. 2016. – separate document

Fig. S1 Phylogenetic relationship of *N. benthamiana* GPAT-related proteins to homologues in *M. truncatula*, *A. thaliana* and *S. lycopersicum*. Individual proteins have been named based on their phylogenetic relationship followed by the unique identifier from their respective genomes (*N. benthamiana* SolGenomics v1.0.1 (Fernandez-Pozo *et al.*, 2015), *A. thaliana* TAIR10 (Berardini *et al.*, 2015), *M. truncatula* Genome Project v4.0 (Bell *et al.*, 2001), *S. lycopersicum* SolGenomics ITAG release 3.10 (Fernandez-Pozo *et al.*, 2015)). Coloured dots indicate species (black = *A. thaliana*, green = *M. truncatula*, red = *N. benthamiana*, blue = *S. lycopersicum*). Numbered brackets indicate major clades (**0** = GPAT9 Clade/outgroup; **1** = GPAT5/7 Clade; **2** = GPAT1/2/3 Clade; **3** = GPAT4/8 Clade; **4** = GPAT6 Clade; **5** = RAM2 Clade).

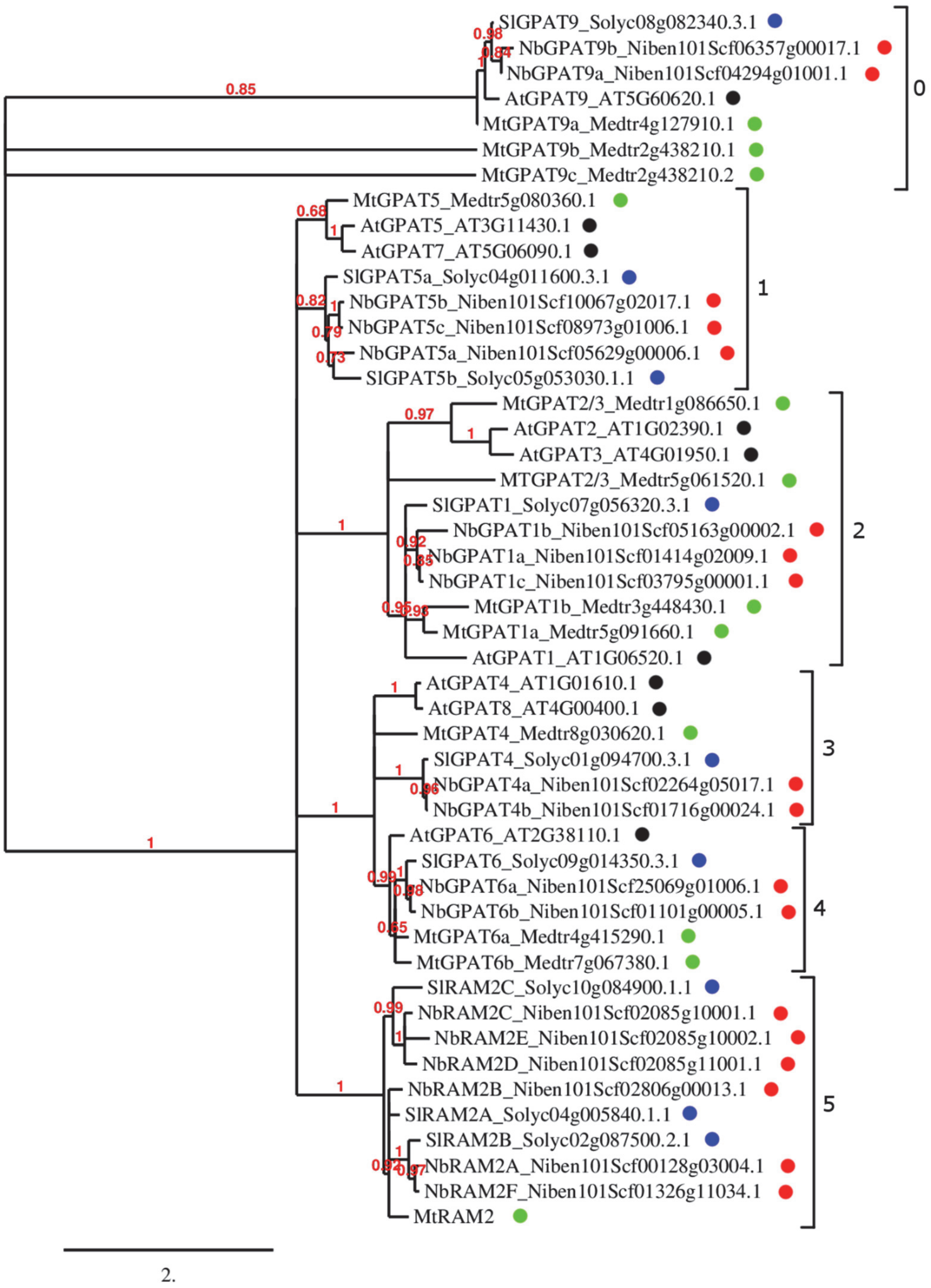

Fig. S2 NbGPAT6a localises to the endoplasmic reticulum and is predicted to have two transmembrane domains. **a.** Subcellular localisation of NbGPAT6a-GFP protein fusion or free GFP upon transient co-expression with ER-RK, an ER-targeted mCherry (Nelson *et al.*, 2007) in *N. benthamiana* leaf epidermal cells (48 h post *Agrobacterium* infiltration). White filled arrowheads indicate the reticulate distribution of NbGPAT6a-GFP. Unfilled arrowheads indicate additional endomembrane structures not labelled by ER-RK. Scale bars represent 10µm. **b.** Probability plot showing the likelihood of inside, outside, or transmembrane helix regions in NbGPAT6a protein. Output generated by TMHMM Server v. 2.0 (Sonnhammer et al., 1998). y-axis shows probability, x-axis shows amino acid positions.

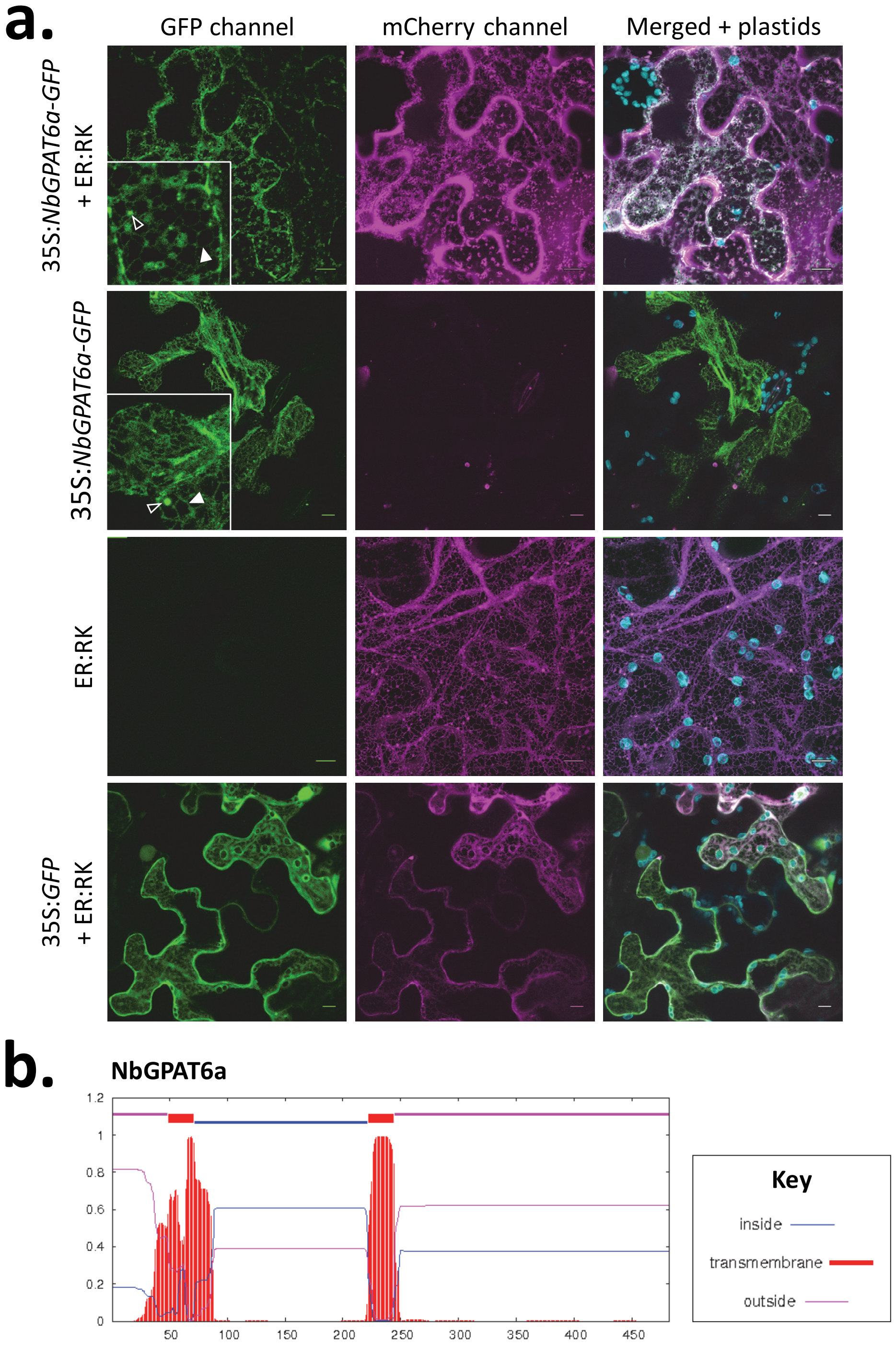

Fig. S3 Cutin content of *NbGPAT6a-GFP* expressing leaves is dramatically increased relative to *GFP 16C* (control) leaves. **a.** Total cutin given as µg/cm^2 leaf area. **b.** Identifiable cutin monomers: FA, fatty acid; DFA, dicarboxylic acid; OH, hydroxyl. Error bars represent standard error of the mean of three biological replicates. Statistical significance was determined using a t-test (*, $p<0.05$; **, $p<0.01$; ***, $p<0.001$).

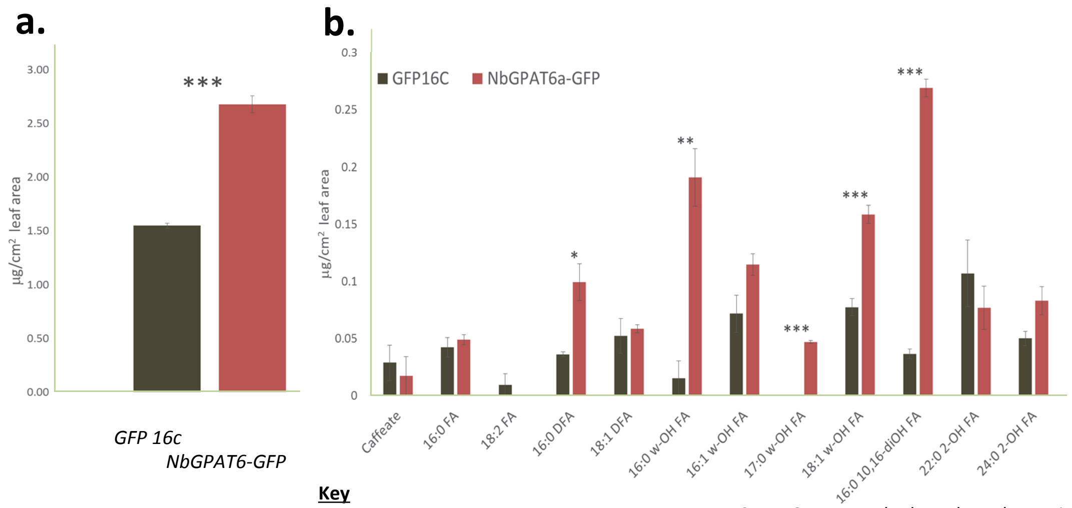

Key

Caffeate = caffeic acid
16:0 FA = hexadecanoic acid
18:2 FA = octadeca-dienoic acid
16:0 DFA = hexadecane-dioic acid
18:1 DFA = octadecene-dioic acid
16:0 ω-OH FA = ω-hydroxy hexadecanoic acid
16:1 ω-OH FA = ω-hydroxy hexadecenoic acid
17:0 ω-OH FA = ω-hydroxy heptadecanoic acid
18:1 ω-OH FA = ω-hydroxy-octadecanoic acid
16:0 10,16-diOH FA = 10,16-dihydroxy hexadecanoic acid
22:0 2-OH FA = 2-hydroxy docosanoic acid
24:0 2-OH FA = 2-hydroxy tetracosanoic acid

Fig. S4 *Phytophthora infestans* haustoria formed in wildtype and NbGPAT6a-GFP overexpressing *Nicotiana benthamiana* leaves are similar in structure. Confocal fluorescence microscopy of detached *Nicotiana benthamiana* leaves stably expressing the indicated genes 2.5 days post infection with *P. infestans* 88069td carrying red fluorescent *tdTomato* gene. Haustoria are indicated by asterisks.

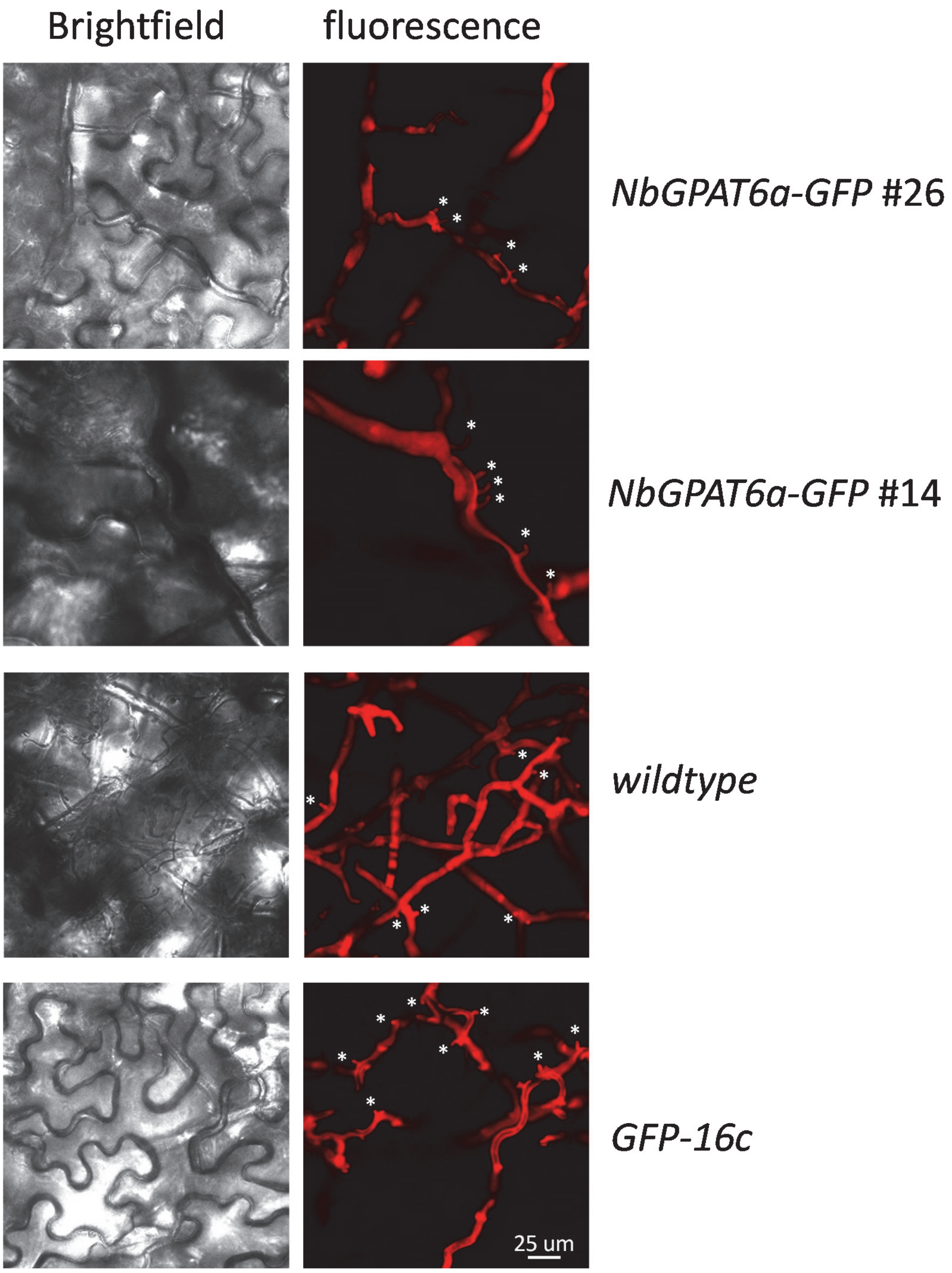

Fig. S5 Transient expression of *NbGPAT6a* does not alter leaf necrosis triggered by *P. infestans* infection. a. Quantification of leaf necrotic area at 5dpi. Pairwise t-test result $p = 0.946$ (n = 25) indicating no significant difference in mean necrotic area between halves of the same leaf infiltrated with either *GFP* or *NbGPAT6a-GFP* constructs. **b.** Representative images of leaves used to quantify necrotic area

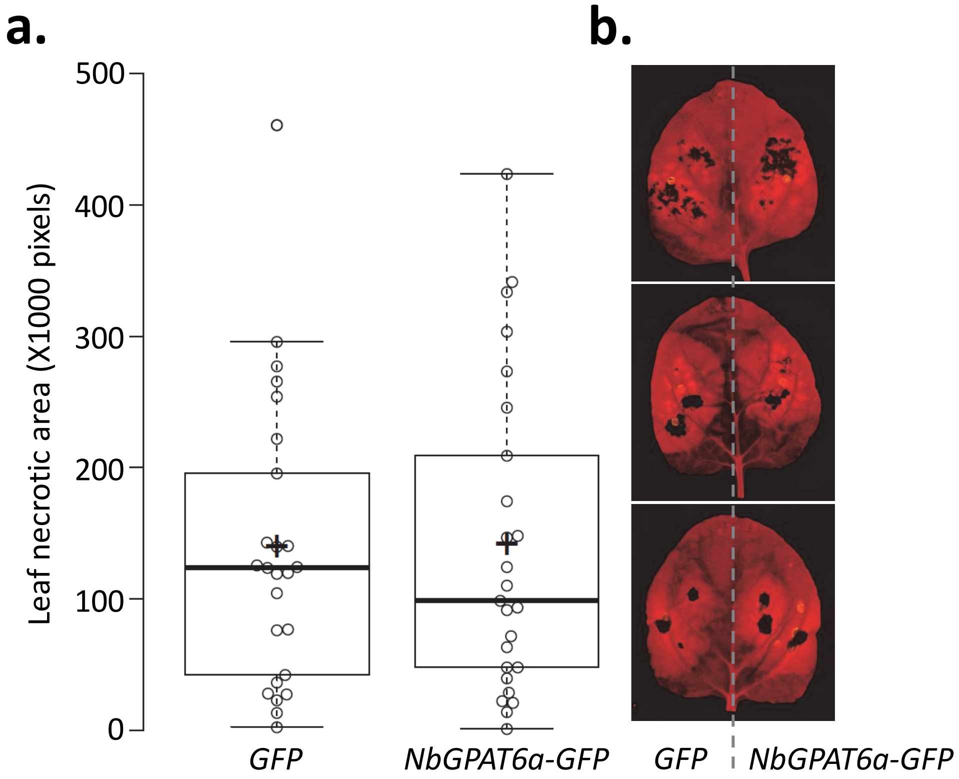

Fig. S6 Assessment of off-target gene silencing following VIGS using siNbGPAT6a or siGUS (control). Relative expression of *NbRAM2* genes with high sequence similarity to *NbGPAT6a* are displayed. Error bars represent standard error of the mean of three biological replicates. Student's t-test used to compare mean relative expression in siNbGPAT6a samples versus highest or lowest in control samples. * = $p < 0.05$ ** = $p < 0.01$ *** = $p < 0.001$.

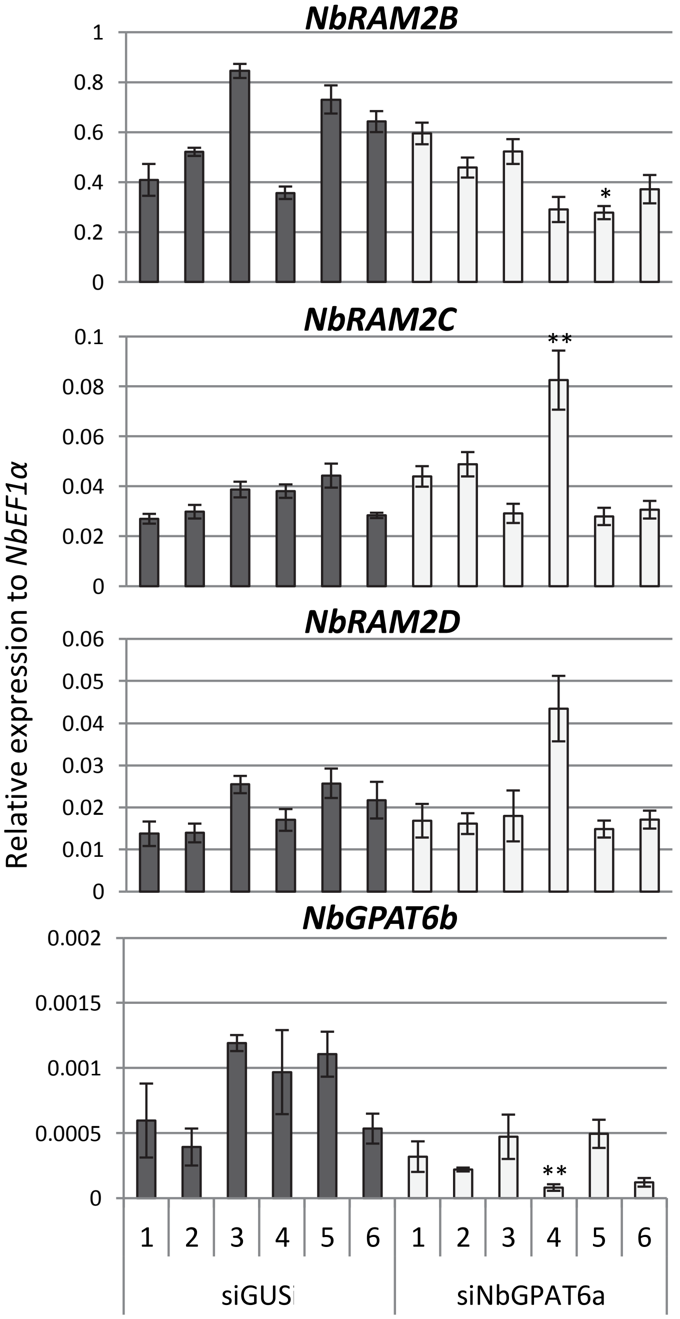

Fig. S7 *Phytophthora infestans* forms digit-like and branched haustoria in tomato *gpat6-a* mutants. Confocal fluorescence microscopy of three detached *gpat6-a* (a.) and two wildtype (b.) tomato cv 'Micro-Tom' leaves 2.5 days post infection with *P. infestans* 88069td carrying red fluorescent *tdTomato* gene. The leaves were taken from separate plants. The merged channel additionally contains chloroplast autofluorescence in magenta. Haustoria are indicated by asterisks. Insets show magnifications of haustoria.

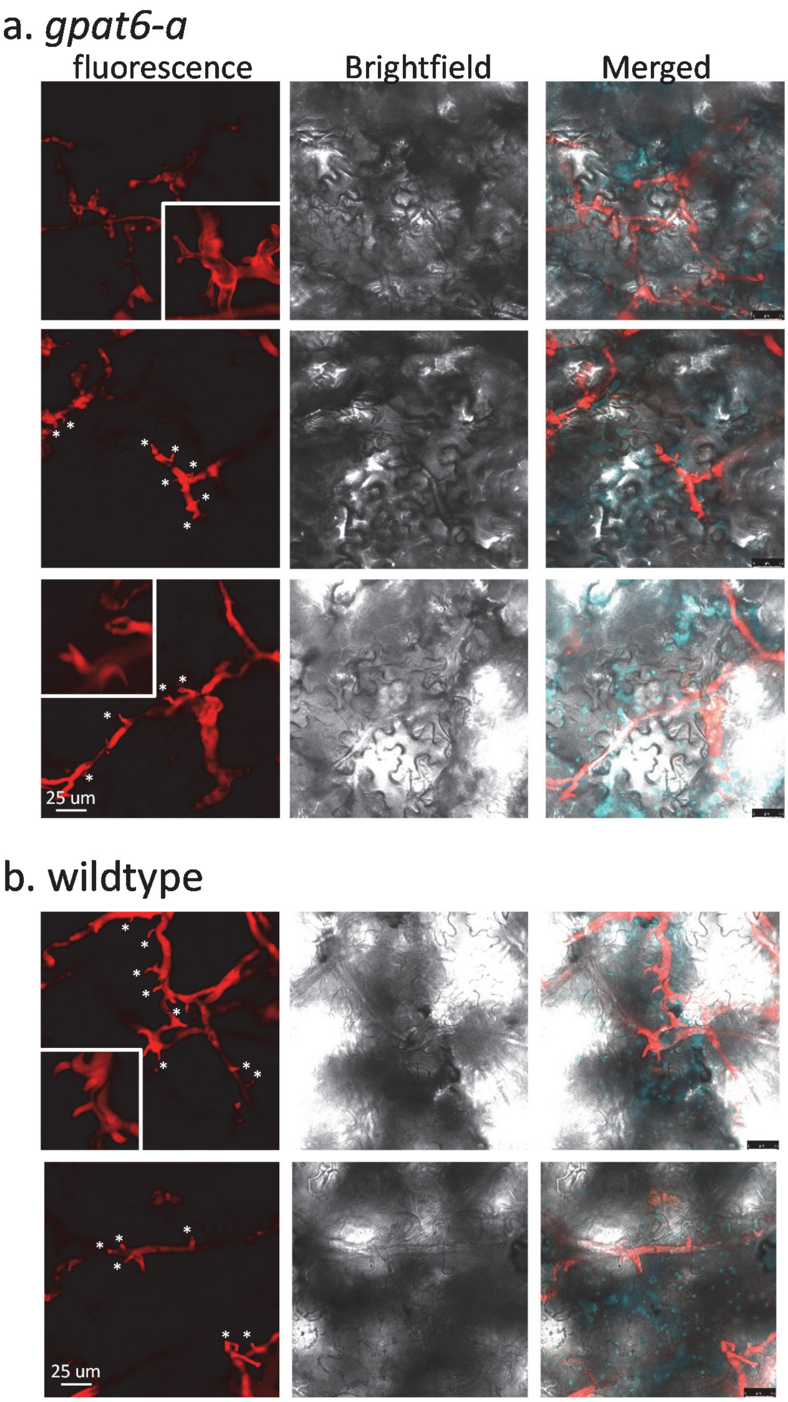

Fig. S8 Thickness of outer cell wall plus cuticle is greater in *gpat6-a* leaves compared to wild-type. **a.** Representative cryo-SEM images of transverse fractures through outer epidermal cell wall and cuticle used to quantify thickness. Scale bars represent 200nm. **b.** Quantification of outer cell wall plus cuticle thickness. Blue circles represent mean of 3 measurements per image (n=6 for WT, n=9 for *gpat6-a*). Error bars represent standard error of the mean. *** = $p < 0.001$. **c.** Representative cross sections of tomato leaf epidermis stained with Toluidine Blue where **c.** shows the upper epidermis, **d.** the lower epidermis. **e.** Wall thickness quantification of both outer and inner periclinal cell walls for upper and lower epidermis based on four images per sample type. * = $p < 0.05$ ** = $p < 0.01$ *** = $p < 0.001$.

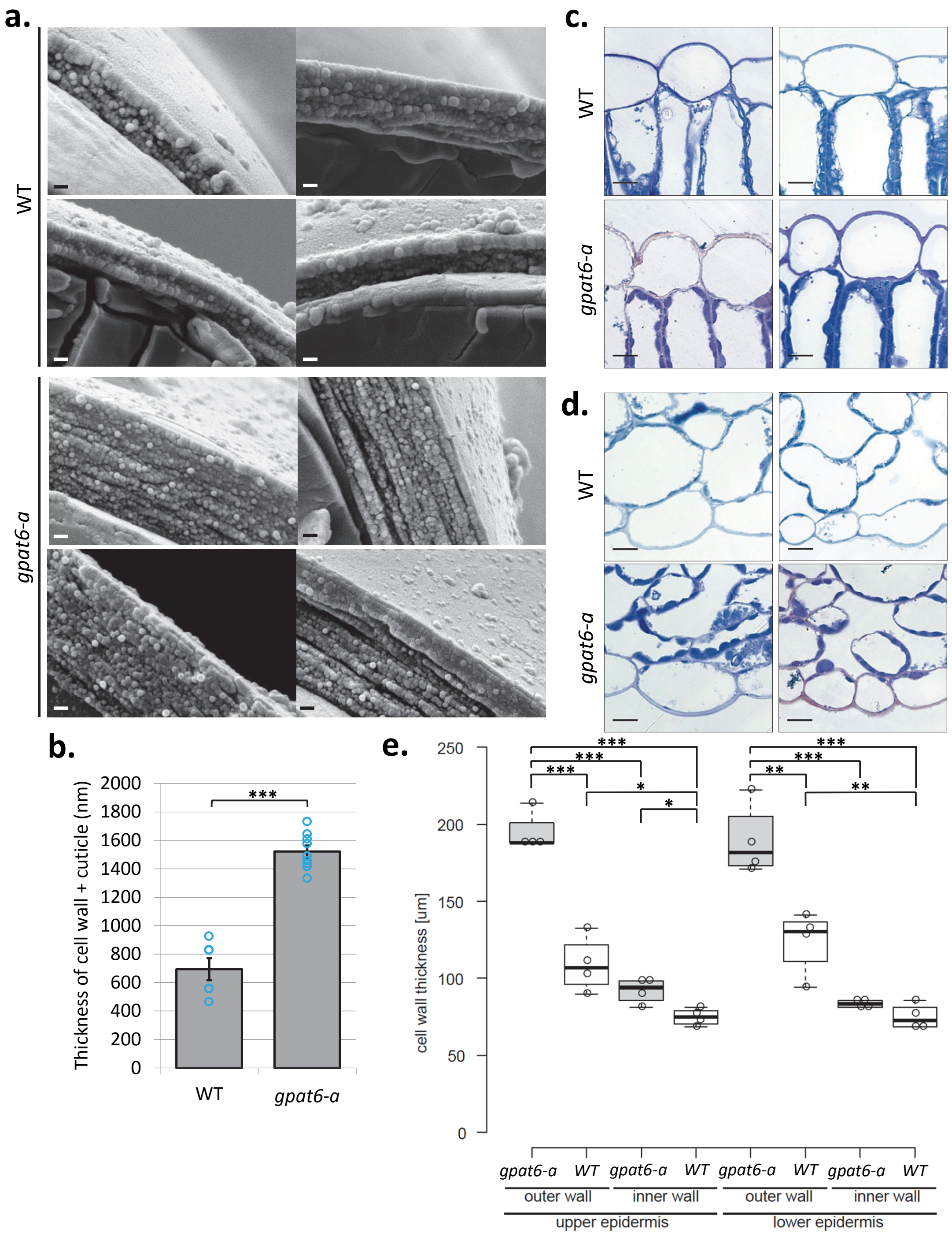

Fig. S9 Leaves of *gpat6-a* tomato mutants are largely impermeable to Toluidine Blue.
a. Leaves were treated with water (left) or abrasives (right, Bentonite/Cellite) and subsequently droplets of Toluidine Blue solution were applied. **b.** Whole leaves were treated on both sides with either cellite/bentonite solution (treated) or water (mock) and then immersed in toluidine blue (0.05%) for 2 minutes. Relative levels of absorbed toluidine blue was measured in total extracts and plotted as absorbance ratio at 630 nm / 435 nm. Error bars represent standard deviation, small letters indicate statistically significant data (t-test).

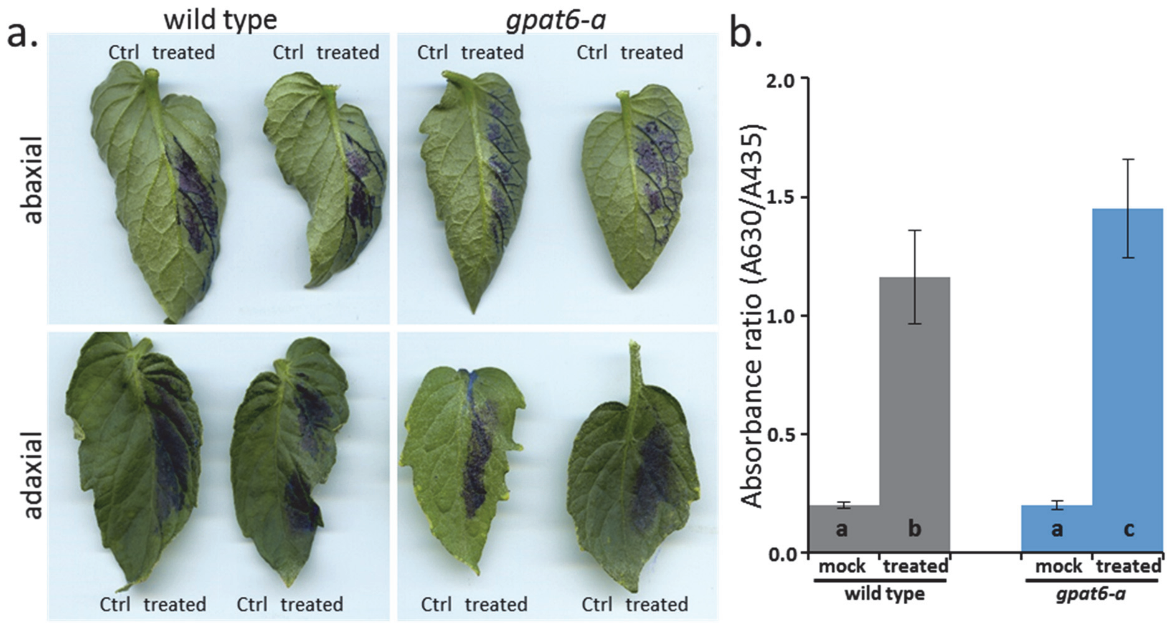

Fig. S10 Tomato *gpat6-a* plants grown in water saturated atmosphere are not altered in their stomata numbers per leaf area. (a) Glass house grown *gpat6-a* plants have reduced numbers of stomata (labelled by asterisks, see Figure 6d for quantification). (b,c) This effect is not observed when plants are grown under water-saturated atmosphere. Scale bar is 100 um for all images. Error bars represent standard deviation from 4 biological replicates.

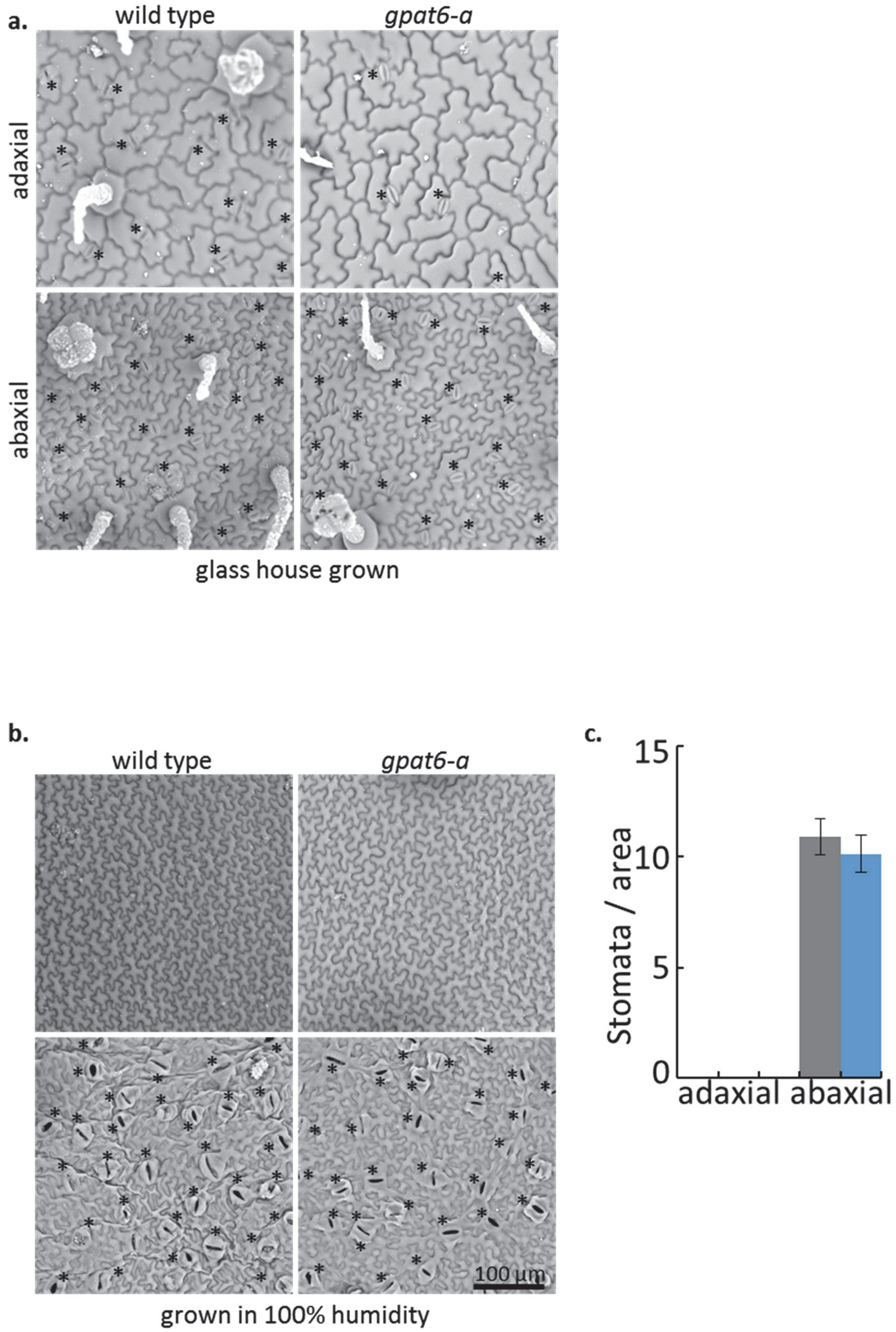

Fig. S11 Stomata numbers and water loss over time remain unaffected in *Nicotiana benthamiana* overexpressing *NbGPAT6-GFP*. Stomatal numbers per leaf are shown (a). Water loss over time (b) and total water loss (c) are shown. Error bars represent standard deviation from 6 biological replicates. The same lowercase letters above error bars indicate no significant difference between means.

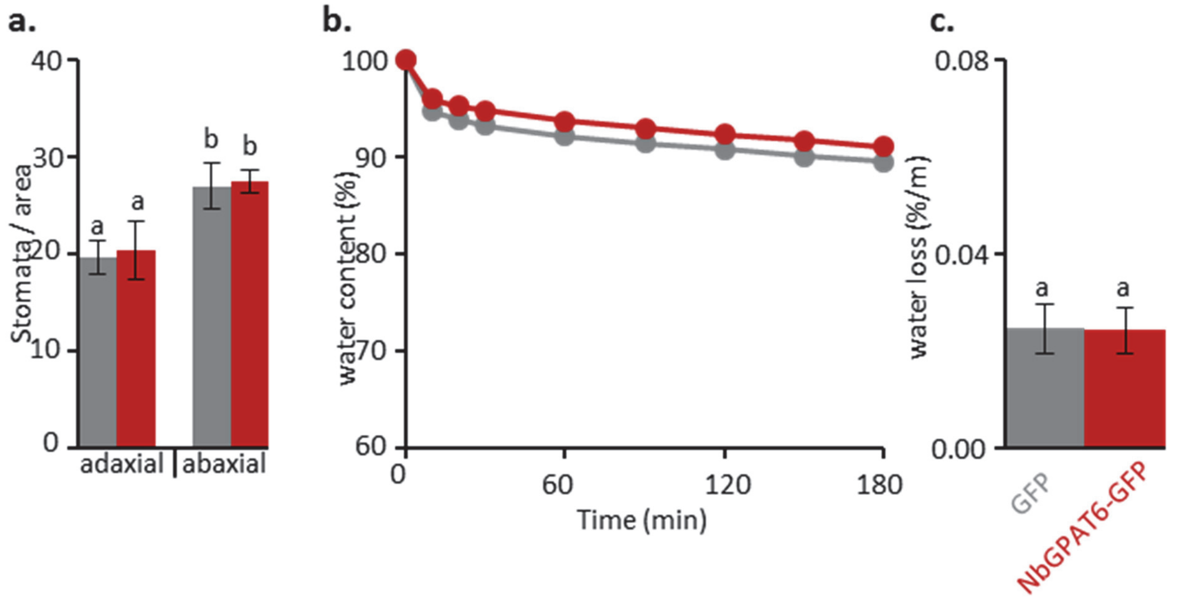

Fig. S12 Composition and overall architecture of the bulk leaf cell wall is not significantly altered in *GPAT6-GFP* overexpressing plants or in *gpat6-a* mutant. Heat map of ELISA absorbance values from cell wall samples (sequentially extracted with CDTA and KOH), screened with a range of monoclonal antibodies towards cell wall components. Values shown are means of 3 biological repeats.

Extract	Line	LM19	LM20	JIM5	JIM7	LM8	LM5	LM26	LM9	LM6-M	LM13	LM16	LM15	LM24	LM25	LM10	LM11	LM23	LM27	LM28	LM21	JIM13	JIM14	JIM15	JIM16	LM2	LM14	JIM4	JIM8
CDTA	*GFP 16C*	1.79	1.42	0.96	1.86	0.04	1.33	0.15	1.92	0.55	0.08	0.13	0.28	0.05	1.25	0.04	0.07	0.05	0.06	0.06	0.04	0.55	0.04	0.06	0.25	1.49	0.20	0.04	0.73
CDTA	*GPAT6a-GFP* #14	2.08	1.62	1.29	2.18	0.04	1.50	0.16	2.05	0.39	0.05	0.13	0.11	0.06	1.08	0.06	0.08	0.06	0.08	0.08	0.04	0.98	0.06	0.09	0.38	1.60	0.26	0.06	1.07
CDTA	*GPAT6a-GFP* #16	1.98	1.71	1.11	2.15	0.04	1.67	0.23	1.95	0.62	0.08	0.14	0.32	0.05	1.34	0.05	0.08	0.07	0.08	0.07	0.05	0.76	0.05	0.08	0.46	1.59	0.21	0.04	0.93
CDTA	*GPAT6a-GFP* #21	1.84	1.45	0.93	1.87	0.04	1.51	0.18	1.78	0.59	0.07	0.10	0.48	0.05	1.46	0.05	0.07	0.06	0.06	0.07	0.04	0.74	0.05	0.07	0.28	1.54	0.25	0.05	0.86
KOH	*GFP 16C*	0.12	0.05	0.07	0.04	0.04	0.06	0.05	0.04	0.04	0.04	0.04	0.50	0.05	1.45	0.05	0.11	0.09	0.09	0.09	0.05	0.05	0.05	0.12	0.05	0.09	0.06	0.06	0.06
KOH	*GPAT6a-GFP* #14	0.18	0.06	0.08	0.05	0.04	0.08	0.07	0.04	0.04	0.04	0.04	0.57	0.07	1.64	0.07	0.11	0.09	0.10	0.10	0.05	0.05	0.05	0.11	0.05	0.11	0.06	0.05	0.06
KOH	*GPAT6a-GFP* #16	0.15	0.05	0.07	0.04	0.04	0.07	0.06	0.04	0.04	0.04	0.04	0.71	0.05	1.61	0.05	0.12	0.09	0.12	0.09	0.05	0.05	0.07	0.12	0.06	0.11	0.06	0.05	0.06
KOH	*GPAT6a-GFP* #21	0.13	0.05	0.07	0.04	0.04	0.09	0.06	0.05	0.04	0.04	0.04	0.51	0.05	1.42	0.05	0.10	0.08	0.09	0.10	0.05	0.05	0.05	0.11	0.05	0.10	0.06	0.05	0.06
CDTA	*wt*	1.92	0.71	0.76	1.62	0.05	1.45	0.38	2.17	1.11	0.23	0.34	0.22	0.05	1.41	0.07	0.24	0.05	0.07	0.12	0.05	0.44	0.05	0.07	0.48	1.38	0.84	0.05	0.40
CDTA	*gpat6-a*	2.05	0.79	0.76	1.72	0.05	1.66	0.43	2.13	1.16	0.28	0.36	0.33	0.05	1.48	0.07	0.28	0.05	0.07	0.13	0.05	0.40	0.05	0.06	0.49	1.34	1.01	0.04	0.39
KOH	*wt*	0.11	0.04	0.07	0.04	0.04	0.05	0.05	0.05	0.04	0.04	0.04	0.26	0.05	1.34	0.08	0.21	0.09	0.09	0.16	0.06	0.06	0.06	0.12	0.06	0.13	0.08	0.05	0.06
KOH	*gpat6-a*	0.12	0.04	0.08	0.05	0.03	0.06	0.06	0.06	0.05	0.04	0.04	0.48	0.05	1.51	0.11	0.25	0.09	0.09	0.19	0.07	0.05	0.05	0.09	0.06	0.11	0.07	0.05	0.06

Homogalacturonan (LM19, LM20, JIM5, JIM7) | xylogalacturonan (LM8) | galactan (LM5, LM26, LM9) | arabinan (LM6-M, LM13, LM16) | Xyloglucan (LM15, LM24, LM25) | xylan (LM10, LM11, LM23, LM27, LM28) | mannan (LM21) | arabinogalactan proteins (AGPs) (JIM13, JIM14, JIM15, JIM16, LM2, LM14, JIM4, JIM8)

Dataset S1 (Supplementary_Dataset_1.xlsx) RNA-seq of *Solanum lycopersicum* Micro-tom WT and *gpat6a* leaves, infected with *Phytophthora infestans*. mRNAs from *Solanum lycopersicum* plants (WT Micro-tom or *gpat6a* Micro-tom) infected with *P. infestans* or mock infected were purified using Poly(A) selection from total RNA sample, and then fragmented. cDNA library preparation was performed with the TruSeq RNA Sample Preparation Kit (Illumina, US) according to the manufacturers protocol. cDNA sequencing of the 3 samples (3 biological replicates) was performed with Illumina NextSeq 2500 in 150 paired end mode. Samples were de-multiplexed and analyzed further.

Methods S1

Transient overexpression of *NbGPAT6a-GFP* in *N. benthamiana* leaves. The *NbGPAT6a* ORF (SolGenomics *N. benthamiana* Genome v1.0.1 Gene ID: Niben101Scf25069g01006.1) was amplified from *N. benthamiana* genomic DNA. It was first cloned into the Gateway entry vector pENTR/D-TOPO and subsequently recombined into pK7FWG2 (Karimi *et al.*, 2013), transformed into competent *Agrobacterium tumefaciens* strain GV3101 cells via electroporation (1500V, 5ms) and grown on LB containing rifampicin/gentamycin/spectinomycin (all 50µg/ml) at 28°C. Overnight grown liquid cultures were centrifuged at 3500 x g for 10 mins and the bacterial pellet resuspended in a solution of 10mM MES and 10mM MgCl2 to an OD600 of 1.0. Acetosyringone was then added at 200µM concentration and the suspension incubated under shaking for a further 2-3 hours at 28°C. Finally, the suspension was infiltrated into the abaxial side of 4 week old *N. benthamiana* leaves using a syringe barrel without needle. For leaf infection assays, leaves were detached 24hrs post-infiltration for immediate inoculation with zoospore or conidiophore suspensions. When performing microscopy to study subcellular localisation of NbGPAT6a-GFP leaves were detached 4 days post infiltration.

Toluidine Blue treatment. Leaves were treated with a mixture of bentonite and cellite (0.02% and 1% w/v, respectively) using cotton wool swabs, or treated with water alone as a control. Leaves were then stained with droplets of Toluidine Blue (TBO, 30 µl, 0.1%) for an hour at room temperature, washed with water and then imaged on a flatbed scanner.
To quantify the extent of TBO staining, a previously published protocol was utilised with modifications (Tanaka *et al.*, 2007). Leaves were treated with cellite/bentonite solution, or mock treated with water and then immersed in toluidine blue (0.05%) for 2 minutes. Leaves were washed in water and leaf disks (8 mm diameter) were excised, ground in 200 µL buffer [200 mM Tris-HCl (pH8.0), 250 mM NaCl, 25 mM EDTA] and 400 µL ethanol was added. Samples were then vortexed, centrifuged, and the supernatant was examined by spectrophotometer for the absorbance at 630 nm (A630) and 435 nm (A435). Relative levels of absorbed TB were calculated as the ratio of A630:A435.

Cutin analysis. One mature leaf from each of three individual 5.5-week old *N. benthamiana* plants expressing GFP16C (control) or NbGPAT6a-GFP was harvested. Images of the leaves generated with a flatbed laser scanner (Epson V30) were analyzed using ImageJ (https://imagej.net) to determine the leaf surface area in order to calculate the amount of cutin per cm^2 leaf area. The leaf samples were then subjected to delipidation using solvents containing 0.01% BHT (butylated hydroxytoluene). For each sample, 30 mL of isopropanol was preheated in a glass vial to 85°C and leaf samples were cut into squares (~0.5 cm^2), added to the hot isopropanol, and incubated at 85°C for 20 minutes. The vials were then cooled to room temperature and agitated at 250 rpm for 1 hour, then the supernatant was poured off. The tissue was then sequentially washed with each of the following solvents: isopropanol (2nd wash), 1:2 methanol: chloroform, 2:1 methanol: chloroform, methanol. A total of 30 mL of solvent was used for each wash, with agitation for at least 2 hours and up to overnight (2:1 methanol: chloroform step). After the final wash, the tissue was dried under a stream of nitrogen gas and then placed in a vacuum desiccator overnight. Cutin depolymerisation was performed as described (Li-Beisson *et al.*, 2013), except that twice the amount of the reaction medium was used and the 60°C incubation step was performed overnight. The final products were filtered through filter paper (VWR #28333-021) into clean vials. An 1 mL aliquot of each cutin sample was dried by heating at 40°C under a stream of nitrogen, then derivatised with 50 µL each of 1 µg/ul pyridine and BSTFA (N,O-bis(trimethylsilyl)trifluoroacetamide) for 10 minutes at 90°C. The samples were dried again by heating to 50°C under nitrogen and re-suspended in 100 µL of

chloroform. The samples were then analysed by gas chromatography (GC) as described (Bolger *et al.*, 2014). Cutin levels were normalised based on the internal standards and the surface area of the leaves (taking into account abaxial and adaxial surfaces).

Cell-wall component enzyme linked immune sorbent assay (ELISA). Leaf material was harvested, frozen, finely ground, and processed into an alcohol insoluble residues (AIR) via sequential washes with 80%, 90%, and 100% (v/v) ethanol, acetone, and finally methanol/chloroform (2:3 v/v) and left to dry overnight. AIR (5 mg) was extracted sequentially with 50 mM 1,2-cyclohexanediaminetetraacetic acid (CDTA), and 4 M KOH. CDTA and KOH samples were diluted 1 in 10 into PBS (KOH samples were neutralised first with glacial acetic acid) and used to coat microtiter plates (maxisorb, Nunc) and ELISAs were performed as described (Torode *et al.*, 2016) using cell wall specific monoclonal antibodies(Cornuault *et al.*, 2017)

Prediction of transmembrane domains used TMHMM Server v. 2.0 (Sonnhammer *et al.*, 1998)

Supporting References

Bell CJ, Dixon RA, Farmer AD, Flores R, Inman J, Gonzales RA, Harrison MJ, Paiva NL, Scott AD, Weller JW, et al. 2001. The Medicago Genome Initiative: a model legume database. *Nucleic Acids Res* **29**(1): 114-117.

Berardini T, Reiser L, Li D, Mezheritsky Y, Muller R, Strait E, Huala E. 2015. The Arabidopsis information resource: Making and mining the "gold standard" annotated reference plant genome. *Genesis* **53**(8): 474-485.

Bolger A, Scossa F, Bolger ME, Lanz C, Maumus F, Tohge T, Quesneville H, Alseekh S, Sørensen I, Lichtenstein G. 2014. The genome of the stress-tolerant wild tomato species Solanum pennellii. *Nature Genetics* **46**(9): 1034.

Cornuault V, Buffetto F, Marcus SE, Crépeau M-J, Guillon F, Ralet M-C, Knox P. 2017. LM6-M: a high avidity rat monoclonal antibody to pectic α-1, 5-L-arabinan. *BioRxiv*: 161604.

Fernandez-Pozo N, Menda N, Edwards JD, Saha S, Tecle IY, Strickler SR, Bombarely A, Fisher-York T, Pujar A, Foerster H, et al. 2015. The Sol Genomics Network (SGN)--from genotype to phenotype to breeding. *Nucleic Acids Res* **43**(Database issue): D1036-1041.

Karimi M, Inzé D, Van Lijsebettens M, Hilson P. 2013. Gateway vectors for transformation of cereals. *Trends Plant Sci* **18**(1): 1-4.

Li-Beisson Y, Shorrosh B, Beisson F, Andersson MX, Arondel V, Bates PD, Baud S, Bird D, DeBono A, Durrett TP. 2013. Acyl-lipid metabolism. *The Arabidopsis book/American Society of Plant Biologists* **11**: 1-65.

Nelson BK, Cai X, Nebenführ A. 2007. A multicolored set of in vivo organelle markers for co‐localization studies in Arabidopsis and other plants. *The Plant Journal* **51**(6): 1126-1136.

Sonnhammer EL, von Heijne G, Krogh A. 1998. A hidden Markov model for predicting transmembrane helices in protein sequences. *Proc Int Conf Intell Syst Mol Biol* **6**: 175-182.

Tanaka H, Watanabe M, Sasabe M, Hiroe T, Tanaka T, Tsukaya H, Ikezaki M, Machida C, Machida Y. 2007. Novel receptor-like kinase ALE2 controls shoot development by specifying epidermis in Arabidopsis. *Development* **134**(9): 1643-1652.

Torode TA, Siméon A, Marcus SE, Jam M, Le Moigne M-A, Duffieux D, Knox JP, Hervé C. 2016. Dynamics of cell wall assembly during early embryogenesis in the brown alga Fucus. *Journal of Experimental Botany* **67**(21): 6089-6100.
